# The Polymycovirus-Mediated Growth Enhancement of the Entomopathogenic Fungus *Beauveria bassiana* Is Dependent on Carbon and Nitrogen Metabolism

**DOI:** 10.3389/fmicb.2021.606366

**Published:** 2021-02-02

**Authors:** Charalampos Filippou, Rebecca M. Diss, John O. Daudu, Robert H. A. Coutts, Ioly Kotta-Loizou

**Affiliations:** ^1^Department of Life Sciences, Imperial College London, London, United Kingdom; ^2^Department of Clinical, Pharmaceutical and Biological Science, University of Hertfordshire, Hatfield, United Kingdom

**Keywords:** fungal growth, fungal sporulation, *Beauveria bassiana*, *Polymycoviridae*, mycovirus

## Abstract

*Polymycoviridae* is a growing family of mycoviruses whose members typically have non-conventional capsids and multi-segmented, double-stranded (ds) RNA genomes. *Beauveria bassiana* polymycovirus (BbPmV) 1 is known to enhance the growth and virulence of its fungal host, the entomopathogenic ascomycete and popular biological control agent *B. bassiana*. Here we report the complete sequence of BbPmV-3, which has six genomic dsRNA segments. Phylogenetic analysis of RNA-dependent RNA polymerase (RdRp) protein sequences revealed that BbPmV-3 is closely related to the partially sequenced BbPmV-2 but not BbPmV-1. Nevertheless, both BbPmV-3 and BbPmV-1 have similar effects on their respective host isolates ATHUM 4946 and EABb 92/11-Dm, affecting pigmentation, sporulation, and radial growth. Production of conidia and radial growth are significantly enhanced in virus-infected isolates as compared to virus-free isogenic lines on Czapek-Dox complete and minimal media that contain sucrose and sodium nitrate. However, this polymycovirus-mediated effect on growth is dependent on the carbon and nitrogen sources available to the host fungus. Both BbPmV-3 and BbPmV-1 increase growth of ATHUM 4946 and EABb 92/11-Dm when sucrose is replaced by lactose, trehalose, glucose, or glycerol, while the effect is reversed on maltose and fructose. Similarly, both BbPmV-3 and BbPmV-1 decrease growth of ATHUM 4946 and EABb 92/11-Dm when sodium nitrate is replaced by sodium nitrite, potassium nitrate, or ammonium nitrate. In conclusion, the effects of polymycoviruses on *B. bassiana* are at least partially mediated *via* its metabolic pathways.

## Introduction

*Polymycoviridae* is a recently established family exclusively accommodating viruses infecting fungi in its sole genus *Polymycovirus*. The first member of the family, *Aspergillus fumigatus* tetramycovirus 1, was reported in 2015 (Kanhayuwa et al., 2015) and since then over 20 related mycoviruses have been fully or partially sequenced (Supplementary Table S1). Polymycoviruses have a variable number of double-stranded (ds) RNA genomic segments, ranging from three (Mu et al., 2018) to eight (Jia et al., 2017; Mahillon et al., 2019), while a closely related, single-stranded (ss) RNA virus with 11 genomic segments named Hadaka virus was recently discovered (Sato et al., 2020). Polymycoviruses are the first dsRNA viruses found to be infectious not only as purified entities but also as naked dsRNA (Kanhayuwa et al., 2015; Jia et al., 2017; Niu et al., 2018); the majority are non-conventionally encapsidated.

*Beauveria bassiana* is an ascomycete belonging to the family *Cordycipitaceae*, order Hypocreales. *B. bassiana* has a widespread geographical distribution and can be found in soil (Garrido-Jurado et al., 2015) and in plants as an endophyte (McKinnon et al., 2017); importantly, *B. bassiana* is an arthropod pathogen with a wide host range and serves as the active ingredient of many popular biopesticides (de Faria and Wraight, 2007). Mycoviruses in general (Herrero et al., 2012; Kotta-Loizou et al., 2015; Koloniuk et al., 2015; Gilbert et al., 2019) and polymycoviruses in particular (Kotta-Loizou and Coutts, 2017; Filippou et al., 2018) have been found to infect *B. bassiana* isolates, in some cases increasing their growth and virulence (Kotta-Loizou and Coutts, 2017) and illustrating potential in biological control applications.

Here we report the complete sequence of *B. bassiana* polymycovirus (BbPmV) 3 and its phylogenetic relationships with members of the *Polymycoviridae* family. Both BbPmV-3 and the previously characterized BbPmV-1 have similar effects on the morphology, sporulation, and growth of their respective host isolates. Polymycovirus-mediated phenotypes are dependent on the constituents of the growth medium, suggesting that polymycoviruses may interfere with carbon and nitrogen metabolism of their host fungus.

## Materials and Methods

### Fungal Isolates and Growth Media

*Beauveria bassiana* isolates EABb 92/11-Dm and ATHUM 4946 originate from Spain and Greece, respectively. The isolates were grown at 25°C, on Potato Dextrose Agar (PDA; Sigma–Aldrich) or Czapek-Dox minimal medium (MM; Sigma–Aldrich) or Czapek-Dox complete medium (CM; MM in addition to 1.5 g/L malt extract, peptone, and yeast extract). For growth assays, the sucrose and sodium nitrate in Czapek-Dox MM were substituted with different carbon and nitrogen sources (Supplementary Table S2; Cai et al., 2018). A cocktail of antibiotics (ampicillin, kanamycin, and streptomycin, each at a final concentration of 100 μg/mL) was used to prevent bacterial contamination. For curing experiments, the protein synthesis inhibitor cycloheximide was added at concentrations up to 1000 μg/mL.

### Growth and Sporulation Assays

Fungal spores from agar plates were collected in phosphate buffered saline (PBS), filtered through Miracloth, and counted using the FastRead counting slides (Immune Systems). The concentration of the fungal spore suspension was adjusted, and 1000 fungal spores were spotted centrally on solid Czapek-Dox CM and growth was monitored for up to 18 days. All experiments were performed in triplicate using three independent stocks for each fungal isolate and statistical analysis was performed using GraphPad Prism 6. Differences in growth were considered to be statistically significant if measurements for at least five consecutive late time points were shown to be statistically significant (*p*-value < 0.05; ANOVA) between virus-infected and virus-free isogenic lines.

### Nucleic Acid Extraction

BbPmV-1 and BbPmV-3 genomic dsRNAs were purified using a small-scale dsRNA extraction procedure. Briefly, total nucleic acids were treated with phenol/chloroform, DNase I (Promega), and S1 nuclease (Promega), and the remaining dsRNA was precipitated with sodium acetate and ethanol. Total fungal RNA and DNA were purified using the RNeasy and DNeasy Plant Mini Kits (Qiagen), respectively, according to the manufacturer’s instructions.

### Reverse Transcription (RT), Polymerase Chain Reaction (PCR), and Molecular Cloning

Random reverse transcription (RT)-polymerase chain reaction (PCR) and RNA ligase mediated rapid amplification of cDNA ends (RLM-RACE) were performed as described by Froussard (1992) and Coutts and Livieratos (2003), respectively. Sequence specific oligonucleotide primers used for RT-PCR include those amplifying the BbPmV-3 RdRp sequence (5′-CCT CAT CTC GCT CAT GTC CC-3′ and 5′-GCA GGC GTA TAG GTC CCT TC-3′) and the universal ITS1F primers (5′-CTT GGT CAT TTA GAG GAA GTA A-3′; Gardes and Bruns, 1993) and ITS4 (5′-TCC TCC GCT TAT TGA TAT GC-3′; White et al., 1990) amplifying the internal transcribed spacer (ITS) sequence. All PCR amplicons were cloned into the pGEM-T Easy vector (Promega) and transformed into *Escherichia coli* XL-10 Gold competent cells (Agilent). Recombinant plasmid DNA was purified using the QIAprep Spin Miniprep Kit (Qiagen). At least three clones for each PCR amplicon were sequenced by Genewiz.

### Computational Analyses

BLASTx analysis (Altschul et al., 1990) using the non-redundant protein database updated on August 2020 was performed to identify sequence similarities. The Pfam database (El-Gebali et al., 2019) was used to identify protein family domains. Sequence logos were generated using WebLogo (Crooks et al., 2004). Intrinsic disorder was predicted using PONDR-FIT (Xue et al., 2010). Maximum-likelihood (ML) phylogenetic analysis was performed using MEGA 6 (Tamura et al., 2013). The sequences were aligned with MUSCLE as implemented by MEGA 6, and all positions with less than 30% site coverage were eliminated. The LG + G + I + F substitution model was used for the RdRP, the putative scaffold protein, and the methyl transferase; the WAG + G substitution model was used for the PASrp. Homologous proteins from the closely related Hadaka virus 1 (Sato et al., 2020) were used as outgroups for the RdRP, the putative scaffold protein, and the methyl transferase; the PASrp from *B. bassiana* non-segmented virus 1 (BbNV-1; Kotta-Loizou et al., 2015) was used as outgroup for the polymycovirus PASrp. The Protein Homology/analogY Recognition Engine v2.0 (Phyre2; Kelley et al., 2015) was used for protein structure predictions. Molecular graphics images were produced using the UCSF Chimera package from the Computer Graphics Laboratory, University of California, San Francisco (supported by NIH P41 RR-01081; Pettersen et al., 2004).

## Results and Discussion

### Sequence Analysis of BbPmV-3

**FIGURE 1:**
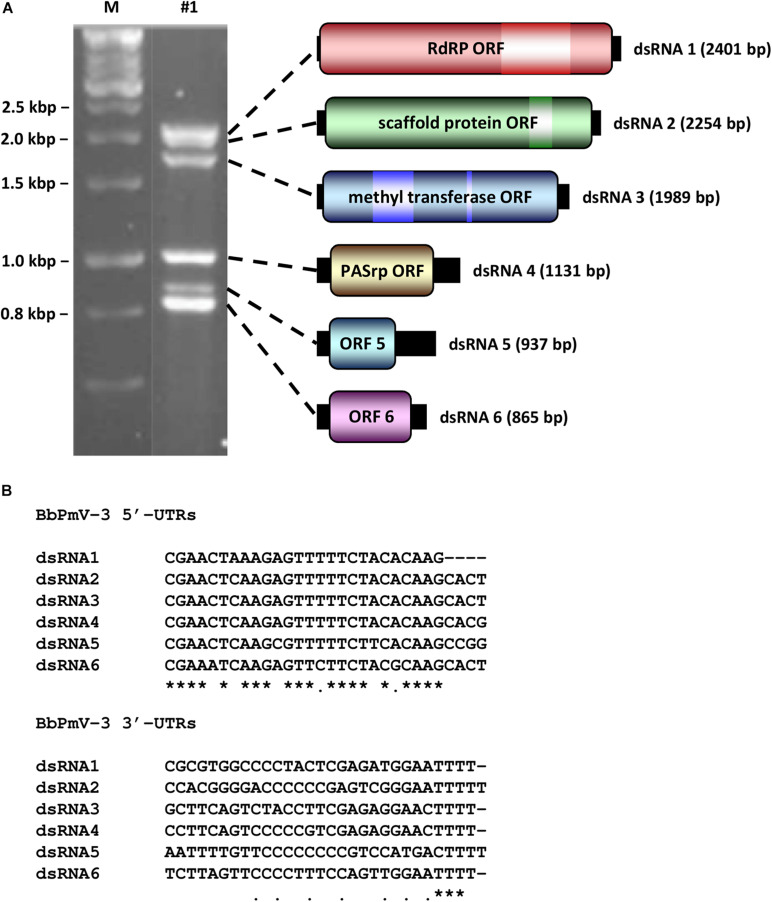
**(A)** Agarose gel electrophoresis (left) and schematic representation (right) of the BbPmV-3 dsRNA genome. The ORFs (dark colored boxes) are flanked by 5′- and 3′-UTRs (black boxes). The light colored box represents known motifs. M indicates DNA marker HyperLadder 1kb (Bioline). **(B)** Alignment of the 5′- and 3′-UTRs of BbPmV-3 dsRNAs 1–6. Asterisks signify identical nucleotides, and dots signify conserved purines or pyrimidines.

BbPmV-3 has the typical genomic organization of other members of the *Polymycoviridae* family (Table 1). The genome of BbPmV-3 comprises six dsRNAs, ranging from 2.5 to 0.9 kbp in length, each one carrying an open reading frame (ORF) flanked by 5′ and 3′ untranslated regions (UTRs; Figure 1A). Both the 5′ and 3′ UTR termini are conserved (Figure 1B), supporting the notion that all six dsRNAs comprise the genome of one single virus. The BbPmV-3 full genomic sequences were submitted to the European Nucleotide Archive (primary accession number PRJEB42287; secondary accession number ERP126123). It should be noted that BbPmV-3 partial sequences corresponding to less than 10% of its genome have been reported previously (Kotta-Loizou and Coutts, 2017) for dsRNAs 1-3 (accession numbers LN896318-LN896320). The first polymycovirus discovered, *Aspergillus fumigatus* tetramycovirus 1, has four genomic segments (Kanhayuwa et al., 2015). Subsequently, related viruses with five (Botryosphaeria dothidea RNA virus 1; Zhai et al., 2016), six (BbPmV 3; Kotta-Loizou and Coutts, 2017), seven (BbPmV 2; Kotta-Loizou and Coutts, 2017), and eight (*Colletotrichum camelliae* filamentous virus 1; Jia et al., 2017; *Fusarium redolens* polymycovirus 1; Mahillon et al., 2019) genomic segments were found. The variability in the number of genomic segments is not a unique feature of the *Polymycoviridae* family; the *Chrysoviridae* family, whose original members also possessed four genomic segments, now accommodates viruses with three to seven genomic segments (Kotta-Loizou et al., 2020).

**TABLE 1:** Properties of BbPmV-3.

Segment	Length (bp)	ORF size			UTR length (bp)		Putative function
		(nt)	(aa)	(kDa)	5′-UTR	3′-UTR	
dsRNA 1	2401	2304	767	83	26	71	RdRP
dsRNA 2	2240	2094	697	74	70	90	Scaffold protein
dsRNA 3	1989	1848	615	66	51	90	Methyl-transferase
dsRNA 4	1131	807	268	29	110	214	PASrp
dsRNA 5	937	513	170	18	101	323	Unknown
dsRNA 6	865	618	205	22	104	143	Unknown

The largest genomic component, dsRNA1, encodes the RNA-dependent RNA polymerase (RdRP) responsible for the replication of the virus. The BbPmV-3 RdRP sequence is most closely related to BbPmV-2 RdRP (identity: 85.53%; E-value: 0.0). Similarly to its homologs in all known polymycoviruses, BbPmV-3 RdRP belongs to the protein family RdRP_1 (PF00680) and has three conserved motifs (Supplementary Figure S1). The GDNQ motif, typically found in negative-sense ssRNA viruses of the order *Monogenavirales*, is conserved in all members of the family *Polymycoviridae*, replacing the GDD motif found in most dsRNA and positive-sense ssRNA viruses (Supplementary Figure S1).

The second largest component, dsRNA2, encodes a protein of unknown function, hypothesized to act as a scaffold protein (Kotta-Loizou and Coutts, 2017). This protein, similarly to all its homologs, contains a conserved N-terminus and a cysteine-rich, zinc finger-like motif (Supplementary Figure S2), and is rich in arginine repeats (R-R, R-X-R), associated with endoplasmic reticulum (ER) retention signals.

The third largest component, dsRNA3, encodes a methyl transferase, responsible for adding a capping structure at the 5′-termini of the positive-sense strands of the viral dsRNAs (Kanhayuwa et al., 2015; Kotta-Loizou and Coutts, 2017). Similarly to other redox enzymes from all kingdoms of life, the polymycovirus methyl transferases are two-domain proteins (Supplementary Figure S3), containing a methyltransferase catalytic motif and an N-terminal Rossmann-fold domain belonging to the protein family methyltransf_25 (PF13649) and the protein clan FAD/NAD(P)-binding Rossmann fold (NADP_Rossmann; CL0063).

The fourth largest component, dsRNA4, encodes a proline-alanine-serine-rich protein (PASrp). BbPmV-3 PASrp is the least enriched in these residues as compared to its homologs, whose PAS content can be up to 32%; however, its PAS content, approximately 22%, is still higher than the UniprotKB average of 20% (Supplementary Figure S4A). The predicted intrinsic disorder in polymycovirus PASrp ranges from 15% for *Magnaporthe oryzae* polymycovirus 1–50% for *Aspergillus spelaeus* tetramycovirus 1, while BbPmV-3 PASrp is 22% disordered (Supplementary Figure S4B). All PASrp have high a pI (Supplementary Figure S4C); with the exception of the *Cladosporium cladosporioides* virus 1 PASrp that has a pI of 7.75, the rest range from 8.37 for *F. redolens* polymycovirus 1 to 9.61 for *A. spelaeus* tetramycovirus 1, while BbPmV-3 PASrp has a pI of 8.94. PASrp is believed to coat the viral RNA genome *in lieu* of a capsid (Kanhayuwa et al., 2015; Zhai et al., 2016; Kotta-Loizou and Coutts, 2017; Niu et al., 2018) and its amino acid composition, intrinsic disorder, and high pI are characteristics that would facilitate protein–RNA interactions. It should be noted that conventional, filamentous particles have been reported for the *C. camelliae* filamentous virus 1 (Jia et al., 2017), which is one of the two known polymycoviruses with eight segments; it is possible that the viral proteins encoded by the additional segments play a role in virion formation.

The two smallest BbPmV-3 dsRNAs, dsRNA5 and dsRNA6, respectively, encode proteins homologous to BbPmV-2 dsRNA6 (identity: 71.76%; E-value: 7e-88) and dsRNA7 (identity: 82.93%; E-value: 6e-111). No other proteins with significant similarity were found in public databases, including those produced by other polymycoviruses or related viruses such as the Hadaka virus (Sato et al., 2020). Typically, polymycovirus proteins produced by RNAs other than the largest four (or in some cases three) do not have any sequence homology or common biochemical properties (Kotta-Loizou and Coutts, 2017); therefore, the clear homology between the smallest dsRNAs of BbPmV-3 and BbPmV-2 indicates that these two viruses are very closely related.

It should be noted that a couple of errors in the sequence of BbPmV-2 dsRNA6 were detected, an additional C at position 487 within the ORF and an additional G at position 738 within the 3′ UTR, where long stretches of, respectively, C and G are located. The correct sequence was confirmed by sequencing three independent clones and the alterations resulted in a predicted protein foreshortened at the C-terminus. Both BbPmV-2 dsRNA6 and BbPmV-3 dsRNA5 possess remarkably long 3′ UTRs, 391 and 323 nt, respectively. BbPmV-2 dsRNA6 encodes a protein 172 aa in length and 18.8 kDa in mass; similarly, BbPmV-3 dsRNA5 encodes a homologous protein 170 aa in length and 18.5 kDa in mass.

### Phylogenetic Analysis of BbPmV-3

**FIGURE 2:**
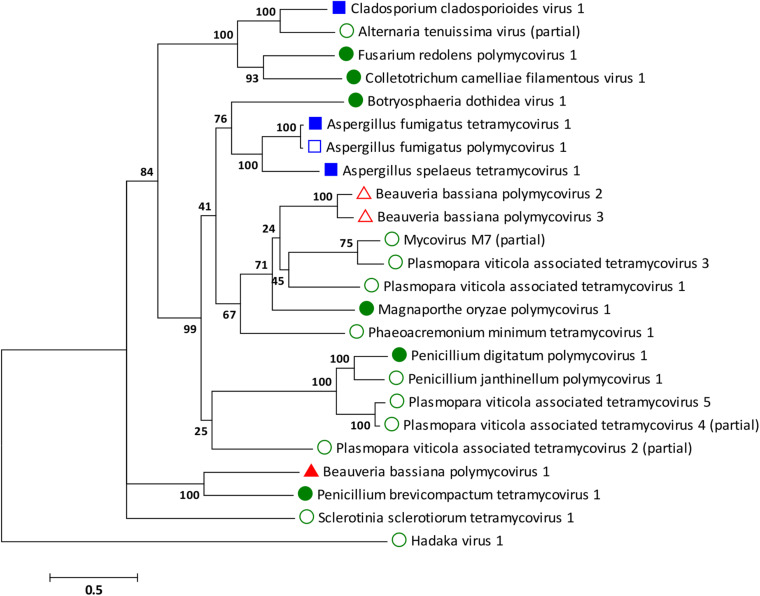
ML phylogenetic tree created based on the RdRP sequences of polymycoviruses. At the end of the branches, established members of the family Polymycoviridae have shapes filled with dark color; other polymycoviruses and related viruses have shape outlines. Blue squares indicate that the virus infects human pathogens; green circles indicate that the virus infects plant pathogens; and red triangles indicate that the virus infects arthropod pathogens.

Phylogenetic analysis was performed for all proteins known to be conserved in members of the family *Polymycoviridae*, the RdRp, the scaffold protein, the methyl transferase, and the PASrp. As expected based on the sequence analysis, the BbPmV-3 RdRp is the closest taxon to the BbPmV-2 RdRp, while the BbPmV-1 RdRp appears to be phylogenetically distant (Figure 2). The distance between BbPmV-3 and BbPmV-1 is supported by the phylogenetic analysis of the scaffold protein (Supplementary Figure S5A), the methyl transferase (Supplementary Figure S5B), and the PASrp (Supplementary Figure S5C). Geographically, ATHUM 4946 harboring BbPmV-3 originated from Athens, Greece; BbPmV-2 has been reported in Syria, Russia, and Uzbekistan (Kotta-Loizou and Coutts, 2017); BbPmV-1 has been found predominantly in Spanish populations (Kotta-Loizou and Coutts, 2017; Filippou et al., 2018).

With the exception of the three polymycoviruses infecting *Aspergillus* spp., there appears to be no correlation between the evolutionary relationships of polymycoviruses and the organism they were isolated from, either in terms of taxonomy, geography, or preferred host. For instance, the five polymycoviruses associated with the oomycete *Plasmopara viticola* do not form a distinct group, but often appear to be more closely related to polymycoviruses infecting ascomycetes than to each other. The majority of polymycoviruses originate from Europe and Asia, with a couple found in Australia and South America (Supplementary Table S1). Most polymycoviruses were isolated from plant pathogens, with *Aspergillus* spp. and *C. cladosporioides* being human pathogens and *B. bassiana* the only arthropod pathogen. Nevertheless, these microorganisms do not have a sole habitat, so contact between them is not unlikely; however, how inter-species transmission of mycoviruses in achieved remains to be elucidated.

### Generation of BbPmV-3-Infected and -Free Isogenic Lines

*Beauveria bassiana* isolate ATHUM 4946 was cured from BbPmV-3 using the protein synthesis inhibitor cycloheximide in combination with single conidia isolation (Supplementary Figure S6A). Elimination of BbPmV-3 was confirmed by RT-PCR using sequence specific oligonucleotide primers designed to generate amplicons 699 bp in length representing a fragment of the coding region of the BbPmV-3 RdRP gene (Supplementary Figure S6B). The identity of the BbPmV-3-infected and -free isolates was confirmed by extracting total DNA and amplifying, cloning, and sequencing the fungal ITS region with ITS specific oligonucleotide primers. Generating virus-free and virus-infected isogenic lines is essential for further phenotypic comparisons, ensuring that observed differences are due to the virus and not the genetic background of the host.

### Effects of Polymycovirus Infection on Fungal Morphology and Sporulation

**FIGURE 3:**
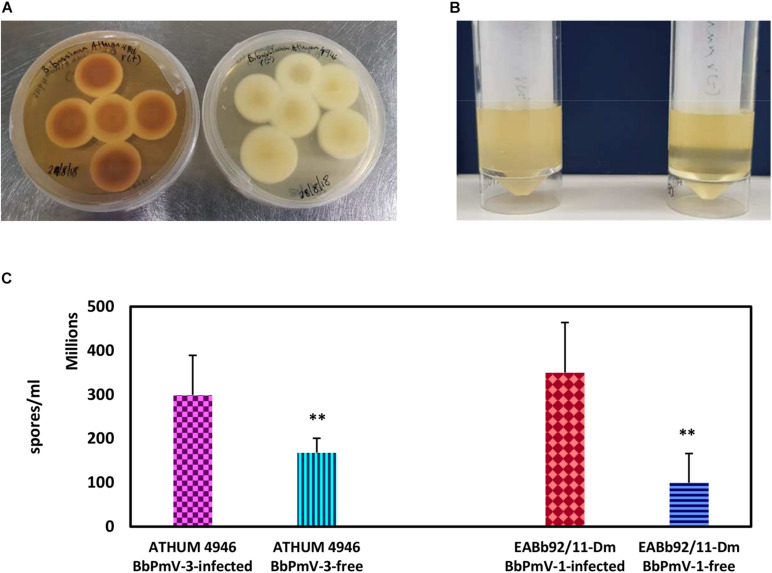
**(A)** Cultures of BbPmV-3-infected (left) and BbPmV-3-free (right) isogenic lines of ATHUM 4649 grown on PDA at 25°C for 2 weeks, showing significant differences in pigmentation. **(B)** Spore suspensions from BbPmV-3-infected (left) and BbPmV-3-free (right) isogenic lines of ATHUM 4649, demonstrating increased sporulation in the BbPmV-3-infected isogenic line as compared to the BbPmV-3-free line. **(C)** Difference in sporulation between virus-infected and virus-free isogenic lines. Student’s *t*-test: ^∗∗^ indicates *p*-value < 0.01.

The morphology of BbPmV-3-infected and BbPmV-3-free isogenic lines was compared after growth on PDA at 25°C for 15 days, showing significant differences in pigmentation (Figure 3A). A less dramatic reduction in pigmentation had been observed previously in BbPmV-1-free *B. bassiana* isolate EABb 92/11-Dm as compared to its respective, virus-infected isogenic line (Kotta-Loizou and Coutts, 2017). Polymycovirus infection has been associated with various morphological alterations, including changes in pigmentation (Kanhayuwa et al., 2015; Kotta-Loizou and Coutts, 2017) and sectoring (Kanhayuwa et al., 2015; Zhai et al., 2016; Kotta-Loizou and Coutts, 2017).

The effects on BbPmV-1 and BbPmV-3 on the sporulation of *B. bassiana* isolates EABb 92/11-Dm and ATHUM 4946, respectively, were assessed. Both virus-infected strains produced approximately twofold more spores as compared to their virus-free isogenic lines, and in both cases, this difference was statistically significant (Figures 3B,C). Increased sporulation, in this case production of asexual conidia, enhances the potential of the fungus and therefore of the polymycovirus to disperse. The effects of polymycovirus infection on sporulation have not been investigated previously; however, other viruses such as *Sclerotinia sclerotiorum* partitivirus 1 (Xiao et al., 2014), *Pseudogymnoascus destructans* partitivirus-pa (Thapa et al., 2016), and uncharacterized dsRNA elements in *Nectria radicicola* (Ahn and Lee, 2001) have been reported to increase sporulation of their fungal hosts. Conversely, Cryphonectria hypovirus 1 (Kazmierczak et al., 1996; Robin et al., 2010), Diaporthe RNA Virus (Moleleki et al., 2003), *Colletotrichum acutatum* partitivirus 1 (Zhong et al., 2014), and two viruses in *Botrytis cinerea* (Potgieter et al., 2013) decrease sporulation of their fungal hosts.

### Effects of Polymycovirus Infection on Fungal Growth

The effects on BbPmV-1 and BbPmV-3 on the growth of *B. bassiana* isolates EABb 92/11-Dm and ATHUM 4946, respectively, were investigated on different carbon and nitrogen sources. The disaccharide sucrose, which serves as the carbon source in Czapek-Dox MM, was replaced by the disaccharides lactose, maltose and trehalose, the monosaccharides fructose and glucose, glycerol, or omitted altogether. Sodium nitrate (NaNO_3_), which serves as the nitrogen source in Czapek-Dox MM, was replaced by sodium nitrite (NaNO_2_), potassium nitrate (KNO_3_), ammonium nitrate (NH_4_NO_3_), ammonium chloride (NH_4_Cl), ammonium sulfate [(NH_4_)_2_SO_4_)], or omitted altogether.

**FIGURE 4:**
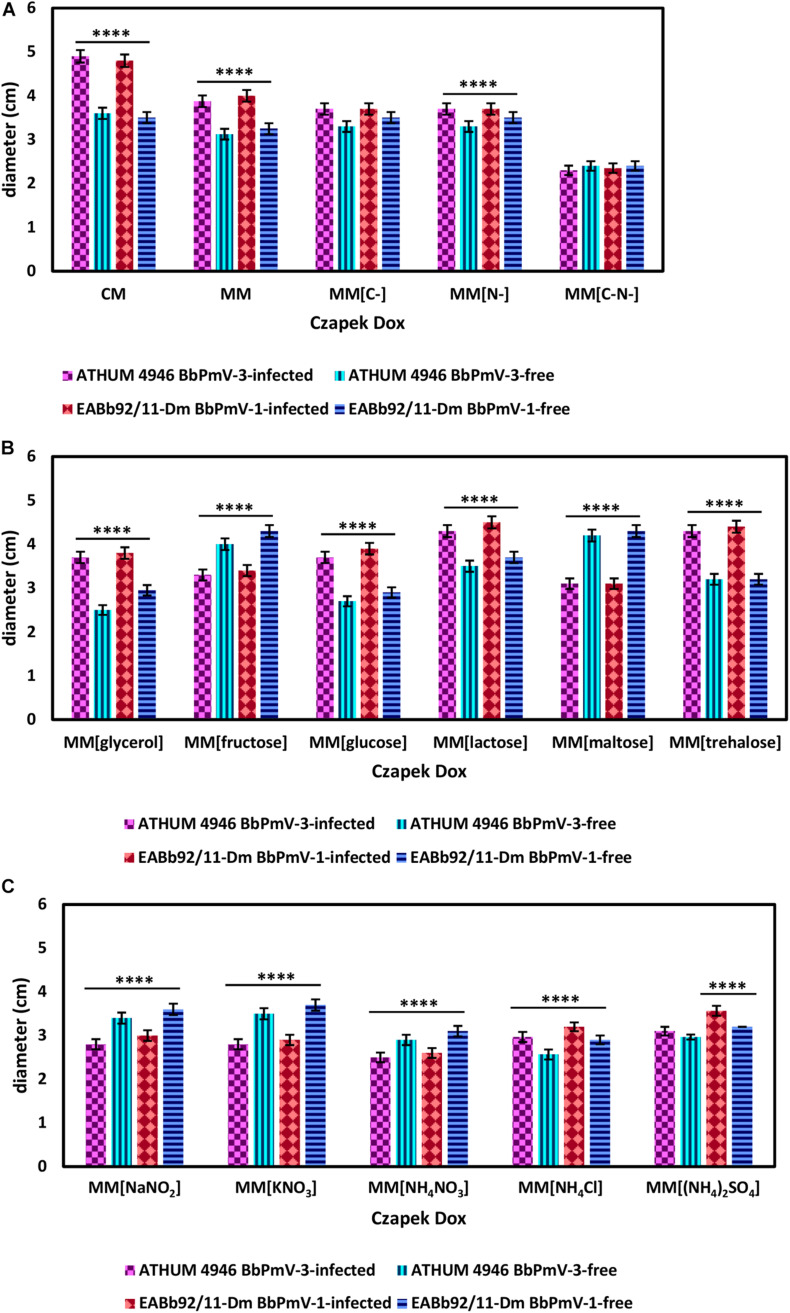
Growth of ATHUM 4946 BbPmV-3-infected and -free (left) and EABb 92/11-Dm BbPmV-1-infected and -free (right) after 18 days on **(A)** Czapek-Dox CM; Czapek-Dox MM; Czapek-Dox MM lacking a carbon source; Czapek-Dox MM lacking a nitrogen source; Czapek-Dox MM lacking both a carbon and a nitrogen source; **(B)** Czapek-Dox MM containing lactose as a carbon source; Czapek-Dox MM containing maltose as a carbon source; Czapek-Dox MM containing trehalose as a carbon source; Czapek-Dox MM containing fructose as a carbon source; Czapek-Dox MM containing glucose as a carbon source; Czapek-Dox MM containing glycerol as a carbon source; **(C)** Czapek-Dox MM containing sodium nitrite as a nitrogen source; Czapek-Dox MM containing potassium nitrate as a nitrogen source; Czapek-Dox MM containing ammonium nitrate as a nitrogen source; Czapek-Dox MM containing ammonium chloride as a nitrogen source; Czapek-Dox MM containing ammonium sulfate as a nitrogen source. Two-way ANOVA; ^*⁣*⁣**^ indicates *p*-value < 0.0001.

BbPmV-3*-*infected ATHUM 4946 and BbPmV-1*-*infected EABb 92/11-Dm demonstrated significantly (*p*-value < 0.05) increased radial growth as compared to their virus*-*free isogenic lines on Czapek-Dox CM and MM (Figure 4A and Supplementary Figures S7, S8A,B), confirming previous observations on EABb 92/11-Dm (Kotta-Loizou and Coutts, 2017). When both a carbon and a nitrogen source were absent, all strains grew very slowly producing very thin mycelium (Supplementary Figure S7) and no significant differences between the virus-infected and the virus-free isogenic lines could be detected (Figure 4A and Supplementary Figure S9C).

When sucrose was replaced by other carbon sources, the virus-mediated increase in growth was maintained in the presence of the disaccharides lactose (Figure 4B and Supplementary Figures S10, S11A) and trehalose (Figure 4B and Supplementary Figures S10, S11C), the monosaccharide glucose (Figure 4B and Supplementary Figures S11, S12B), and glycerol (Figure 4B and Supplementary Figures S10, S12C). Conversely, the BbPmV-3*-*infected ATHUM 4946 and BbPmV-1*-*infected EABb 92/11-Dm grew significantly (*p*-value < 0.05) slower as compared to their virus*-*free isogenic lines in the presence of the disaccharide maltose (Figure 4B and Supplementary Figures S10, S11B) and the monosaccharide fructose (Figure 4B and Supplementary Figures S10, S12A). The virus-mediated effect on growth disappeared when a carbon source was absent (Figure 4A and Supplementary Figures S7, S9A). Since glucose is a direct substrate for glycolysis, the first step of respiration, and all other sugars need to be catabolized and/or modified to be utilized, it is possible that the polymycoviruses affect a metabolic process downstream of glycolysis.

It should be noted that trehalose in particular is the major carbohydrate in the insect hemolymph (Thompson, 2003), and is considered a growth-promoting factor in the case of entomopathogenic fungi such as *B. bassiana* (Pendland et al., 1993). The BbPmV-1-infected EABb 92/11-Dm strain grows significantly (*p*-value < 0.0001) faster on trehalose as compared to sucrose (Table 2). Fungi have evolved two mechanisms for trehalose utilization: (1) secretion of trehalases that hydrolyze extracellular trehalose into glucose, followed by uptake and assimilation of the resultant glucose, and (2) direct uptake of trehalose *via* active transport and subsequent intracellular catabolism. *B. bassiana* encodes homologs of AGT1, a glucoside transporter found in *Saccharomyces cerevisiae*, which is implicated in germination, vegetative growth, and conidial yield on various carbohydrate carbon sources (Wang et al., 2013).

**TABLE 2:** Comparison of growth on different carbon sources.

	MM[lactose] *vs* MM	MM[maltose] *vs* MM	MM[trehalose] *vs* MM	MM[fructose] *vs* MM	MM[glucose] *vs* MM	MM[glycerol] *vs* MM
BbPmV-3*-*infected ATHUM 4946	NS	↓↓↓↓	NS	↓↓↓↓	↑	NS
BbPmV-3*-*free ATHUM 4946	NS	↑↑↑↑	NS	↑↑↑↑	NS	↓↓↓↓
BbPmV-1-infected EABb 92/11-Dm	↑↑↑↑	↓↓↓	↑↑↑↑	↓↓↓	↑↑↑↑	NS
BbPmV-1-free EABb 92/11-Dm	↑↑↑↑	↑↑↑↑	NS	↑↑↑↑	↑↑	NS

*Two-way ANOVA; | *p*-value < 0.05; | | *p*-value < 0.01; | | | *p*-value < 0.001; | | | | *p*-value < 0.0001.*

The opposite phenotype in the case of maltose and fructose is due to both a significant (*p*-value < 0.0001) growth increase of the virus-free strains and a significant (*p*-value < 0.001) growth decrease of their virus-infected isogenic lines (Table 2). This may be attributed to potential effects of polymycoviruses on the metabolic pathway prior to the conversion of these sugars to glucose, such as the alpha/beta-glycosidase encoded by the *agdC* gene that cleaves the alpha(1,4)glycosidic bond of maltose to yield molecules of glucose.

The BbPmV-3*-*infected ATHUM 4946 and BbPmV-1-infected EABb 92/11-Dm grew significantly (*p*-value < 0.05) slower as compared to their virus*-*free isogenic lines when sodium nitrate was replaced by other nitrogen sources (Figure 4C and Supplementary Figure S13), such as sodium nitrite (Supplementary Figure S14A), potassium nitrate (Supplementary Figure S14B), ammonium nitrate (Supplementary Figure S14C), ammonium chloride (Supplementary Figure S15A), ammonium sulfate (Supplementary Figure S15B), or omitted altogether (Figure 4A and Supplementary Figures S7, S9B). In most cases, the growth of virus*-*free ATHUM 4946 and EABb 92/11-Dm is the same on alternative nitrogen sources (Table 3). Conversely, the majority of virus*-*infected isogenic lines grow consistently slower (*p*-value < 0.0001) on any other nitrogen source as compared to sodium nitrate (Table 3). Moreover, it is evident (Figure 4 and Supplementary Figures S14, S15) that any virus-mediated effects, either positive or negative, on fungal growth are more striking in the presence of nitrate and nitrite salts; the presence of ammonium salts lessens these effects that may even become non-significant (e.g., Supplementary Figure S15B). Nitrate is converted to nitrite and then to ammonia/ammonium, which can be used for amino acid biosynthesis. Therefore, it is likely that polymycoviruses specifically affect the uptake and/or the assimilation of nitrate salts. Remarkably, the opposing effects on fungal growth in the presence of two different nitrate salts, sodium nitrate (Supplementary Figure S8B) and potassium nitrate (Supplementary Figure S14B), indicate that the polymycovirus-mediated phenotypes may be pleiotropic and that effects may be exerted at different control points of metabolic pathways.

**TABLE 3:** Comparison of growth on different nitrogen sources.

	MM[NaNO_2_] *vs* MM	MM[KNO_3_] *vs* MM	MM[NH_4_NO_3_] *vs* MM	MM[NH_4_Cl] *vs* MM	MM[(NH_4_)_2_SO_4_] *vs* MM
BbPmV-3*-*infected ATHUM 4946	↓↓↓↓	↓↓↓↓	↓↓↓↓	↓↓↓↓	↓↓↓↓
BbPmV-3*-*free ATHUM 4946	NS	NS	NS	↓↓↓↓	NS
BbPmV-1-infected EABb 92/11-Dm	↓↓↓↓	↓↓↓↓	↓↓↓↓	↓↓↓↓	NS
BbPmV-1-free EABb 92/11-Dm	↑↑	↑↑↑↑	NS	NS	NS

*Two-way ANOVA; | *p*-value < 0.05; | | *p*-value < 0.01; | | | *p*-value < 0.001; | | | | *p*-value < 0.0001.*

The growth trends illustrated by ATHUM 4946 and EABb 92/11-Dm on the various media are similar but not identical, suggesting that the fungal isolates themselves differ in their genetic background. ATHUM 4946 and EABb 92/11-Dm are infected with different polymycoviruses BbPmV-3 and BbPmV-1, respectively, which have the ability to modulate host metabolic pathways in a similar but not identical way. The observed variation may be attributed to the fungal hosts, the polymycoviruses, the specific host–virus pairs under study, the presence of a second virus BbNV-1 in EABb 92/11-Dm (Kotta-Loizou et al., 2015), and/or a combination of these factors.

The number of mycoviruses that enhance fungal growth and/or virulence is increasing in the literature (Ahn and Lee, 2001; Özkan and Coutts, 2015; Thapa et al., 2016; Kotta-Loizou and Coutts, 2017; Aihara et al., 2018; Okada et al., 2018; Shah et al., 2018, 2020); however, the majority of mycoviruses are known to cause no obvious phenotypic changes or a debilitating effect on their host fungus. In plant pathogenic fungi in particular, such as Cryphonectria parasitica, mycovirus-mediated hypovirulence has been successfully utilized in biological control applications (Rigling and Prospero, 2018). There is accumulating evidence that the ability of the mycoviruses to confer a specific phenotype to their fungal host is conditional and this has been clearly illustrated in the case of two betachrysoviruses: Alternaria alternata chrysovirus 1 downregulates growth in vitro and increases virulence in planta (Okada et al., 2018), while M. oryzae chrysovirus 1 strain A modulates pathogenicity depending on the rice variety (Aihara et al., 2018). Our present work on polymycoviruses further supports this notion, indicating that BbPmV-1 and -3 interfere with basic *B. bassiana* metabolic pathways.

## Data Availability Statement

The original contributions presented in the study are publicly available. These data can be found here: https://www.ncbi.nlm.nih.gov/PRJEB42287.

## Author Contributions

IK-L and RC conceived the project. IK-L designed the experiments. CF, RD, and JD performed the experiments. IK-L analyzed the data and wrote the manuscript. RC edited the manuscript. All authors contributed to the article and approved the submitted version.

## Conflict of Interest

The authors declare that the research was conducted in the absence of any commercial or financial relationships that could be construed as a potential conflict of interest.
