# Supplementary material for: The Polymycovirus-Mediated Growth Enhancement of the Entomopathogenic Fungus Beauveria bassiana Is Dependent on Carbon and Nitrogen Metabolism
Source: Front Microbiol. 2021 Feb 2;12:606366. doi: 10.3389/fmicb.2021.606366 (PMC7884332; doi:10.3389/fmicb.2021.606366)
Supplement: Supplementary file 1 [file Data_Sheet_1.PDF]

Supplementary Material

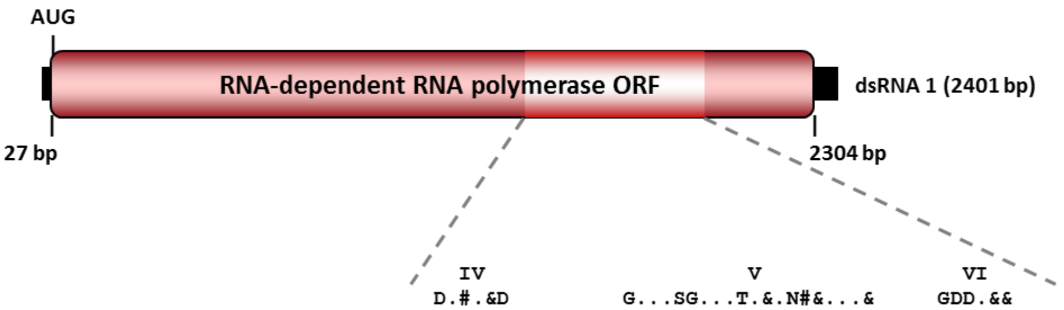


```
                                                IV              V                  VI
                                              D.#.&D      G...SG...T.&.N#&...&    GDD.&&

Beauveria bassiana polymycovirus 3             MSDIQKWDASIV[54]YGTMPSGSYYTSLINTNANNL[35]YGDNQLFSSAL

Alternaria tenuissima virus                    MSDIAKWDANMS[54]YGCMPSGSFYTSLLNTVGNDL[36]YGDNQLVFSSL
Aspergillus fumigatus polymycovirus 1          MSDITKWDANMC[54]YGCMPSGSFYTSLVNTTGNNL[35]YGDNQLFSEHL
Aspergillus fumigatus tetramycovirus 1         MSDITKWDANMC[54]YGCMPSGSFYTSLVNTTGNNL[35]YGDNQLFSEHL
Aspergillus spelaeus tetramycovirus 1          MSDIYKWDANMV[54]FGCMPSGSFYTSLVNTTGNNL[35]YGDNQLFSEEL
Beauveria bassiana polymycovirus 1             MSDIEKWDANMS[54]YGCMPSGSFYTSLLNTVANTL[32]YGDNQLIITSL
Beauveria bassiana polymycovirus 2             MSDILKWDASVV[54]YGTMPSGSYYTSLINTNANNL[35]YGDNQLFSTVL
Botryosphaeria dothidea virus 1                MSDIEKWDANMC[54]SGCMPSGSYYTSLINTIGNDL[35]YGDNQLISEEL
Cladosporium cladosporioides virus 1           MSDIAKWDANMS[54]FGCMPSGSFYTSCVNTIGNDL[35]YGDNQLIFDSL
Colletotrichum camelliae filamentous virus 1   MSDIAKWDANMC[54]FGCMPSGSYYTSMLNTVANDM[35]YGDNQLIFEEL
Fusarium rendolens polymycovirus 1             MSDIAKWDANMP[54]FGCMPSGSFYTSLLNTIGNDI[35]YGDNQLIFESL
Magnaporthe oryzae polymycovirus 1             MSDIDKWDGNVR[54]YGSMPSGSYYTSLVNTNANNL[35]YGDNQLFSEYL
Penicillium brevicompactum tetramycovirus 1    MSDIQKWDANMS[54]FGCMPSGSFYTSLLNTIANDM[32]YGDNQLIIDEL
Penicillium digitatum polymycovirus 1          MSDIEKWDANMS[54]YGCMPSGSYYTSLLNTVGNNL[35]YGDNQIVGEEL
Phaeoacremonium minimum tetramycovirus 1       MSDIEKWDANFP[54]YGCMPSGSFYTSFVNTEGNNL[35]YGDNQLFSERI
Plasmopara viticola associated tetramycovirus 1 MSDIDKWDASMR[54]YGTMASGSYHTSVTNTNANNL[35]YGDNQIFSEKL
Plasmopara viticola associated tetramycovirus 2 MTDISKWDANMS[54]FGCMPSGSYYTSLLNTIANDL[34]YGDNQLIAERM
Plasmopara viticola associated tetramycovirus 3 MSDIWKWDSFIR[54]YGAMPSGSYYTSLLNTNANNL[35]YGDNQLFSARM
Plasmopara viticola associated tetramycovirus 4 ------------[24]YGCMPSGSYYTSPLNTLGNNL[35]YGDNQIVSEEL
Plasmopara viticola associated tetramycovirus 5 MSDIAKWDAYMC[54]YGCMPSGSYYTSPLNTLGNNL[35]YGDNQIVSEEL
Sclerotinia sclerotiorum tetramycovirus 1      MSDIEKWDASAT[54]HGTMPSGSYYTSLVNTVCNDL[32]YGDNQLFSTAM
Hadaka virus 1                                 MADIKHWDTALV[54]KGCMGSGSFFTSPVNTDANSA[29]NGDNQLASSEL
                                               *:** :**        * * ***:.**  **  *        ****:    :
```

Fig. S1. Schematic representation of BbPmV-3 dsRNA1, encoding an RdRp whose ORF (dark red coloured box) is flanked by 5′- and 3′-UTRs (black boxes). The light red coloured box represents the RdRP_1 protein family and a multiple alignment of all known polymycoviruses and related viruses illustrates the conserved RdRp motifs IV to VI.

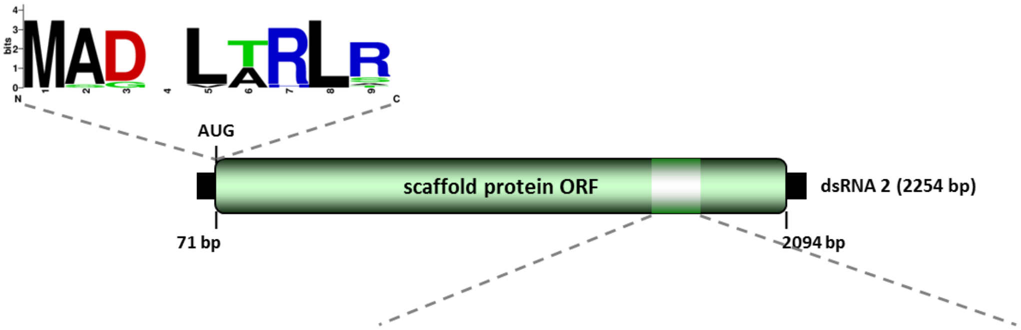


```
Beauveria bassiana polymycovirus 3             APTTAMFMAATEVLLP-PVCHLHS-RGDPAVFLDTAGLVDGQCDYCVGFSRAASAVLSMMTD

Alternaria sp. FA0703 sequence                 RLTLPIPFYSTEVLLP-PACH-HIVDMDPDEYLDDFGTGNPNCFQCEAHYRALSAVGDALDR
Aspergillus fumigatus polymycovirus 1          TPDVNVVYACAEALLP-PLCG-HGLELGPDDYLQCASLVDEDCPACNAHYRATAVLSAACAD
Aspergillus fumigatus tetramycovirus 1         TPDVNVVYACAEALLP-PLCG-HGLELGPDDYLQCASLVDEDCPACNAHYRATAVLSAACAD
Aspergillus spelaeus tetramycovirus 1          TAQVPINFACAEALIP-PLCP-HGAQAPLEEYLDTSSLIDEECPNCNAHYRSVALLSAACAK
Beauveria bassiana polymycovirus 1             DAPLKCILFSTDLIIP-PACP-HCETVDLDHFLASRGVLSEDCRECTVFGRALRLMSELMST
Botryosphaeria dothidea virus 1                SERSNVLMGVAEALLP-PLCD-HAADVSVDDYLDSLSQVEEDCKACEAHYRSVVLLSTLCVR
Cladosporium cladosporioides virus 1           LASG--SFFCGDILIP-DVCR-HPPDGT----------DDECLACANFDYSVFTVGDVCQL
Colletotrichum camelliae filamentous virus 1   TIDV--VFQCSDVLFP-SVCE-HQFDLGERAFDSL--EVGVLCSSCQSFEGVVALVEDALAV
Fusarium rendolens polymycovirus 1             TFKP--GFFCSDILIP-DLCA-HKFVAAEESFVDP--TVGGDCPSCQEFQYVLAVLEESLSV
Magnaporthe oryzae polymycovirus 1             IADPPPLLYTSEILLP-PFCV-HSYQCTAEALLETGGLVDDTCDQCELVSRACAVLSSILDR
Melampsora lini sequence                       IPKERVQFITSEFIIPM-PCE-VHLEEDPEQFLSSFGRIEQECNDCLRYHVAQMSLLSFTGD
Penicillium brevicompactum tetramycovirus 1    SVGIPNALSTVDILLP-PSCY-HWPATPVDTMVDTFGDVDESCMSCQDSVRALRRTAELLSV
Penicillium digitatum polymycovirus 1          VLSGALTFGTADFILP-NLCAAHARRSEVAHAIECNGAFEYTCHNCVGFAHGLRDTVELVGM
Phaeoacremonium minimum tetramycovirus 1       SEQSSVLFATAEFLLP-PLCN-HGLHSPVDVYLSSFSQVADDCAMCDSHYRALSILMGPIVD
Plasmopara viticola associated tetramycovirus 5 VSALTTVFGTCDFILP-SMCGIHARRSTRDRALETNGVFEAGCHQCETFGRGLRDVVELISM
Sclerotinia sclerotiorum tetramycovirus 1      QMALPVRFFSSEILLPVPTCP-HA-GAPFDSFVRAKGALVSGCASCTGVKSAITMVYSLLKT
Hadaka virus 1                                 TGDVKMSYASVDLLFP-DACE-HLLSRAIGDDIP-----ETSCSRCLQ-RRRIMDMVDGLQQ
                                                          : ::*   *                      *  *
```

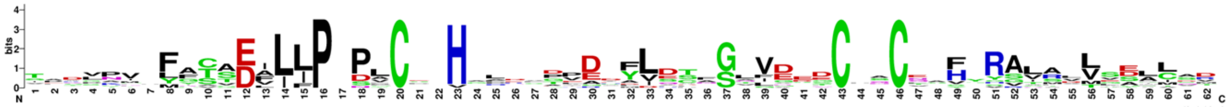

Fig. S2. Schematic representation of BbPmV-3 dsRNA2, encoding a putative scaffold protein whose ORF (dark green coloured box) is flanked by 5′- and 3′-UTRs (black boxes). The light green coloured box represents the cysteine-rich zinc finger, and a multiple alignment of all known polymycoviruses and related viruses illustrates the conserved cysteine and proline residues. Sequence logos for the conserved N-terminus and the cysteine-rich zinc finger of the putative scaffold protein were also generated.

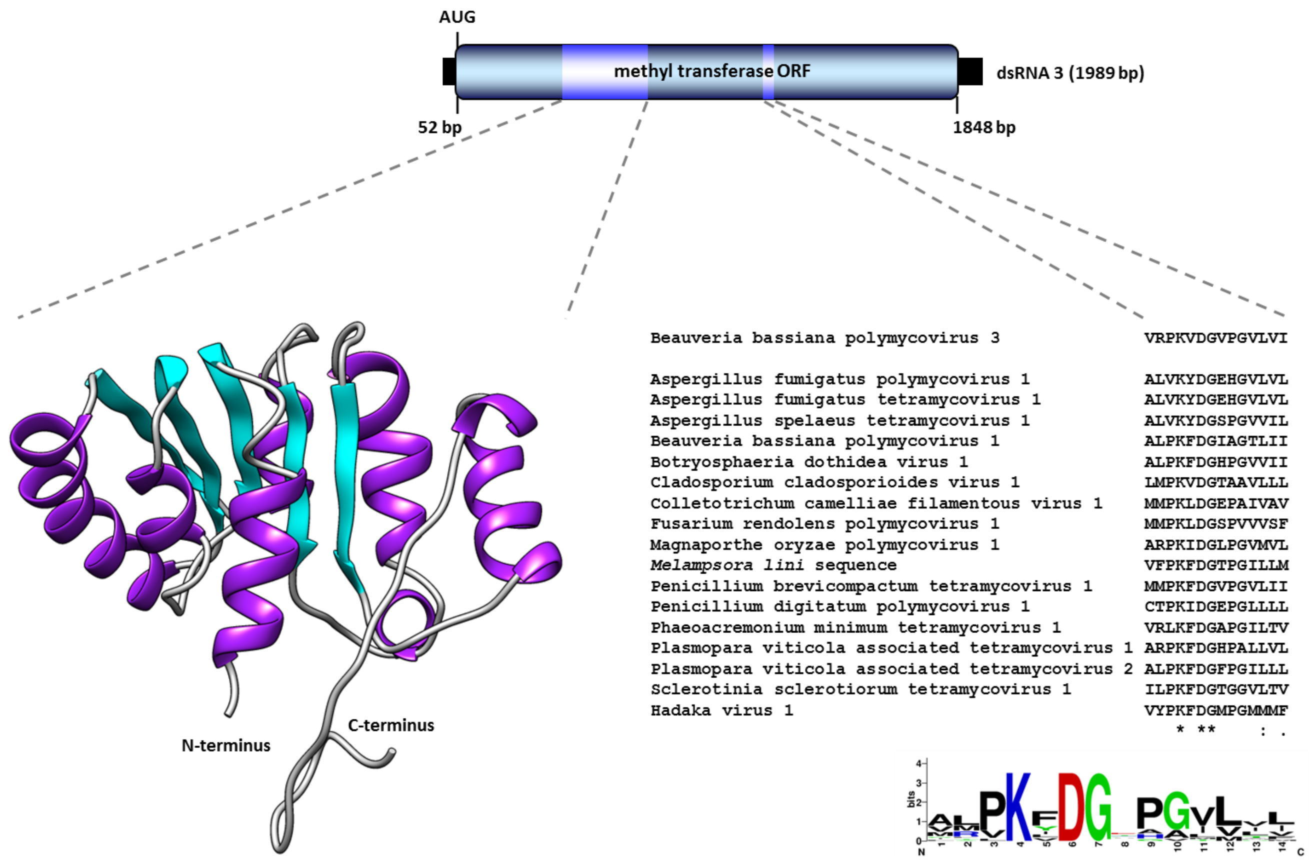

Fig. S3. Schematic representation of BbPmV-3 dsRNA3, encoding a methyl transferase whose ORF (dark blue coloured box) is flanked by 5′- and 3′-UTRs (black boxes). The light blue coloured boxes represent the methyltransf_25 protein family and the methyl transferase catalytic motif. The structure of Rossmann fold domain where the methyl transferase cofactor binds is visualized. A multiple alignment of all known polymycoviruses and related viruses illustrates the conserved catalytic residues of the methyl transferase, for which a sequence logo was also generated.

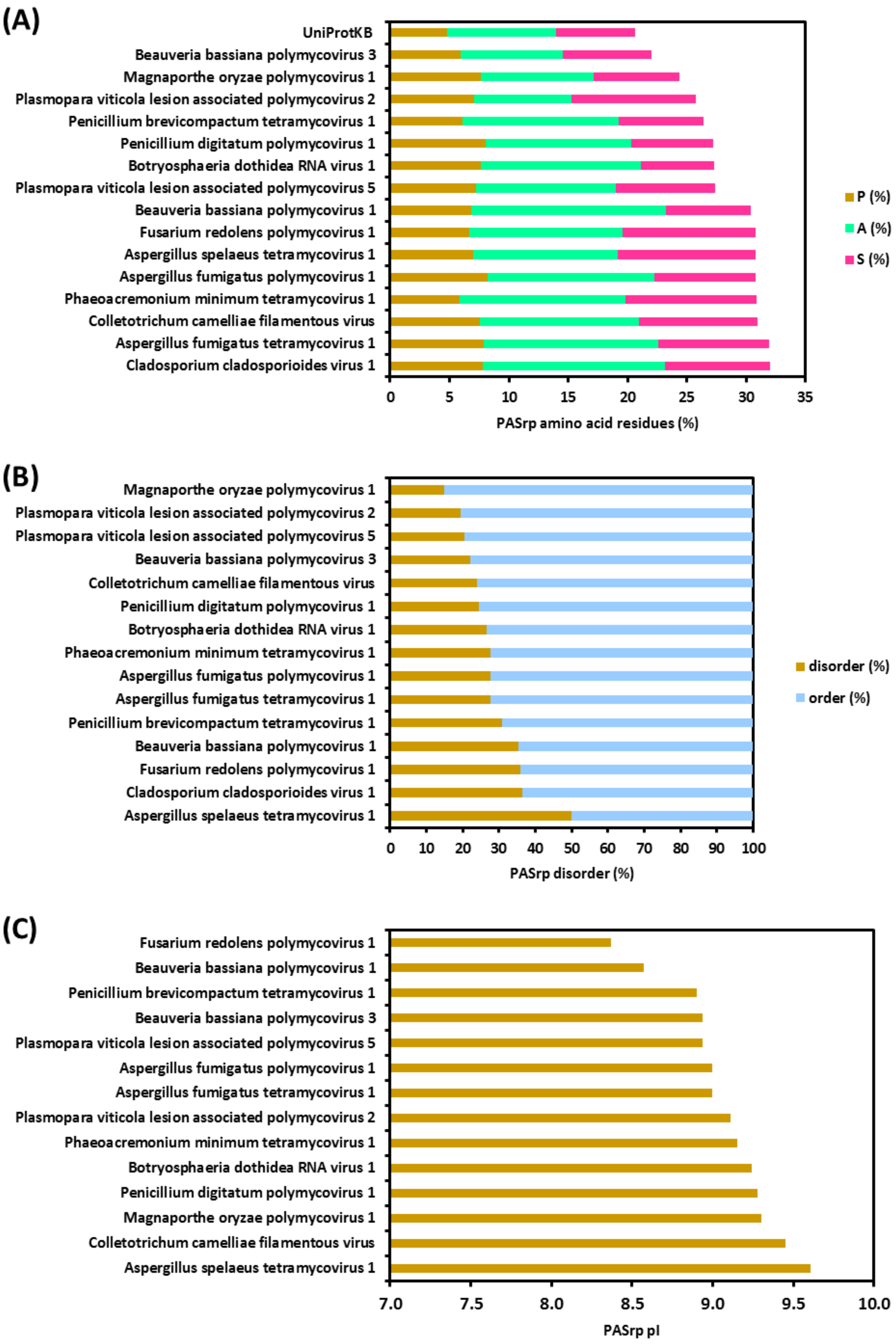

Fig. S4. (A) Amino acid composition, **(B)** predicted intrinsic disorder and **(C)** pI of polymycovirus PASrp.

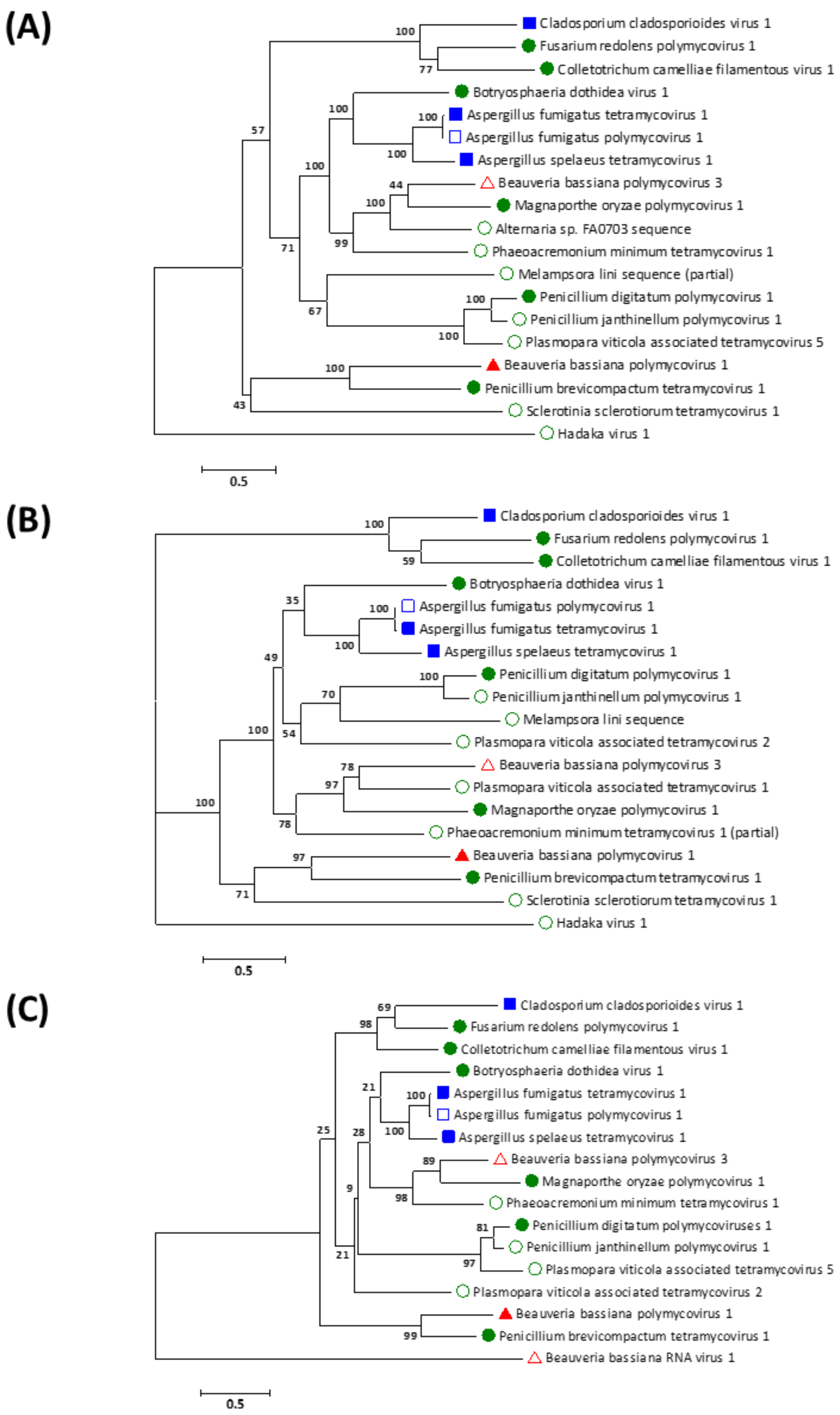

Fig. S5. ML phylogenetic tree created based on the sequences of **(A)** the putative scaffold protein; **(B)** the methyl transferase; **(C)** the PASrp. At the end of the branches, established members of the family *Polymycoviridae* have shapes filled with dark colour; other polymycoviruses and related viruses have shape outlines. Blue squares indicate that the virus infects human pathogens; green circles indicate that the virus infects plant pathogens; red triangles indicate that the virus infects arthropod pathogens.

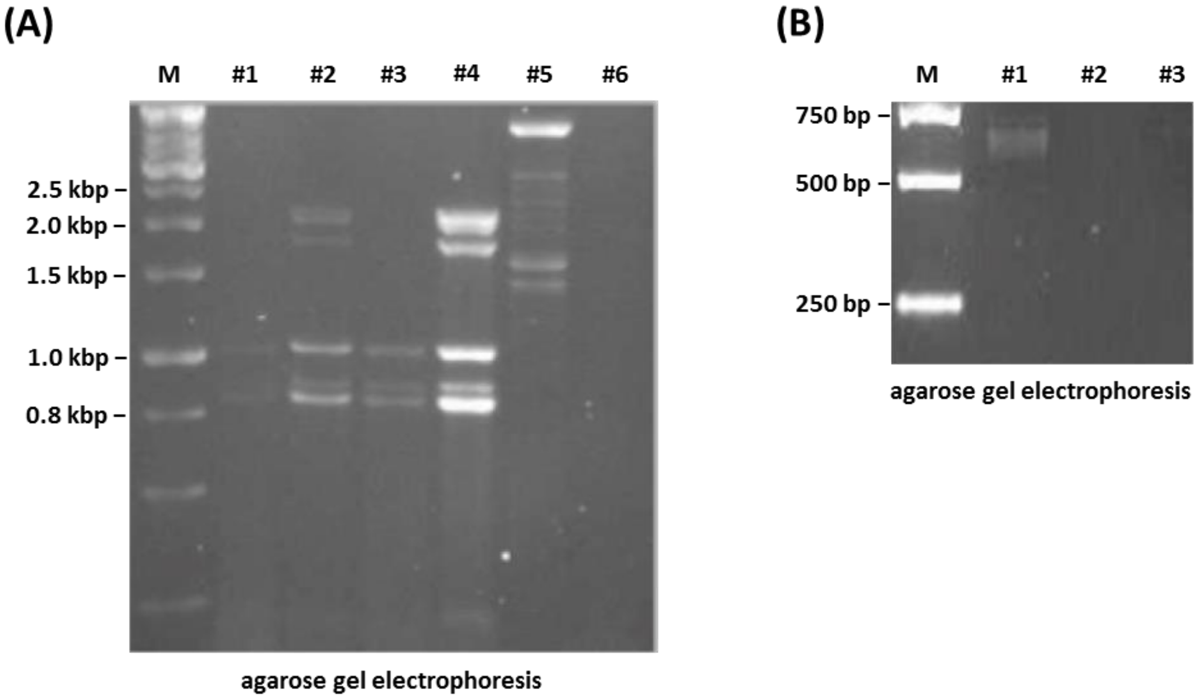

Fig. S6. (A) BbPmV-3 dsRNAs extracted from ATHUM 4946 derived from single colonies after cycloheximide treatment. Lanes #1-#4 are uncured replicates of ATHUM 4946 while lane #6 contains a cured isogenic line of ATHUM 4946. Lane #5 contains a positive control dsRNA isolated from the *B. bassiana* IMI 392612 strain. **(B)** RT-PCR amplicons (699 bp) representing a fragment of the BbPmV-3 RdRP coding region (lane #1), absent from the negative control (water, lane #2) and a cured, BbPmV-3–free isogenic line (lane #3).

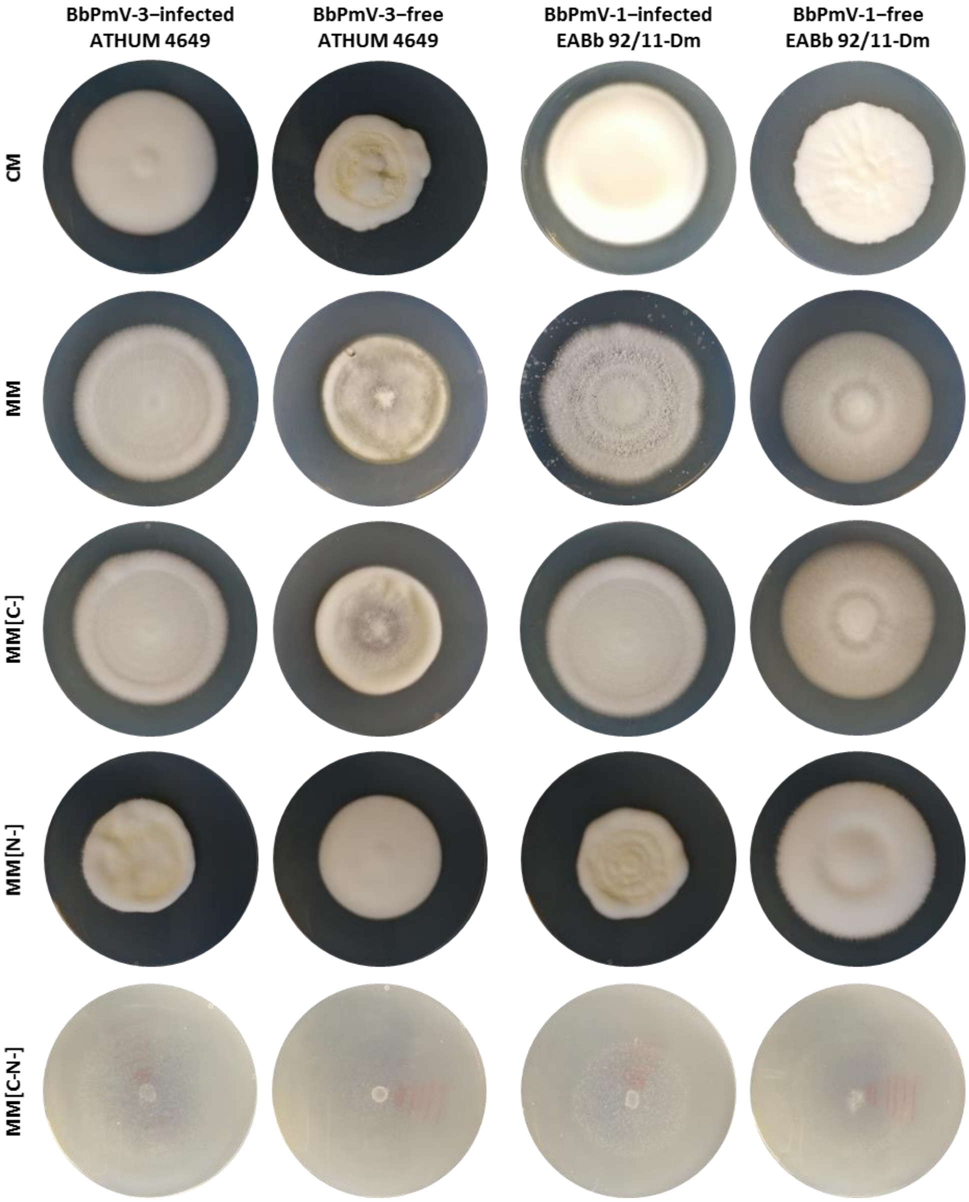

Fig. S7. Cultures of ATHUM 4946 BbPmV-3–infected and –free (left) and EABb 92/11-Dm BbPmV-1–infected and –free (right) on Czapek-Dox CM; Czapek-Dox MM; Czapek-Dox MM lacking a carbon source; Czapek-Dox MM lacking a nitrogen source; Czapek-Dox MM lacking both a carbon and a nitrogen source.

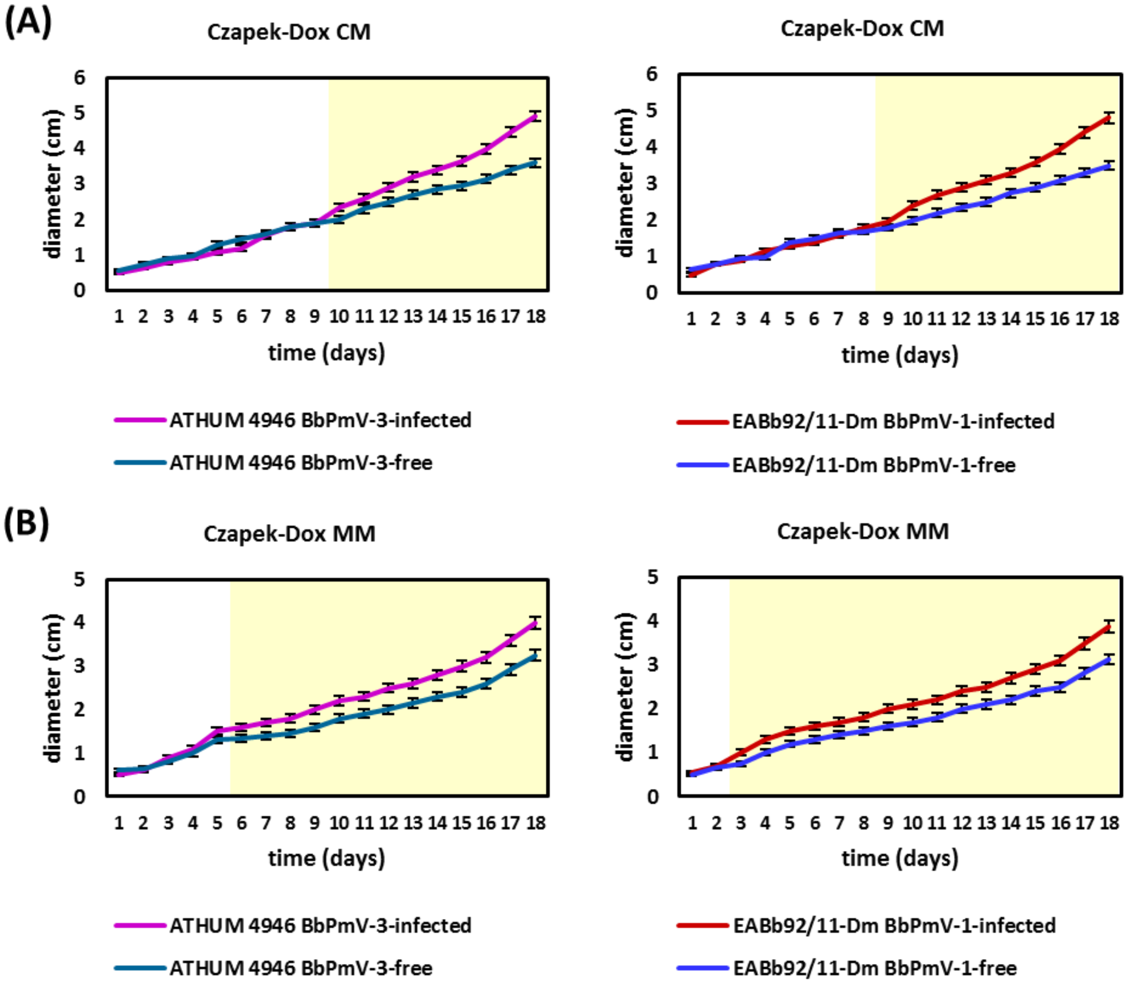

Fig. S8. Growth curves of ATHUM 4946 BbPmV-3–infected and –free (left) and EABb 92/11-Dm BbPmV-1–infected and –free (right) on **(A)** Czapek-Dox CM and **(B)** Czapek-Dox MM. Light yellow background indicates a statistically significant difference between the isogenic lines (2-way ANOVA; P-value < 0.05 at least).

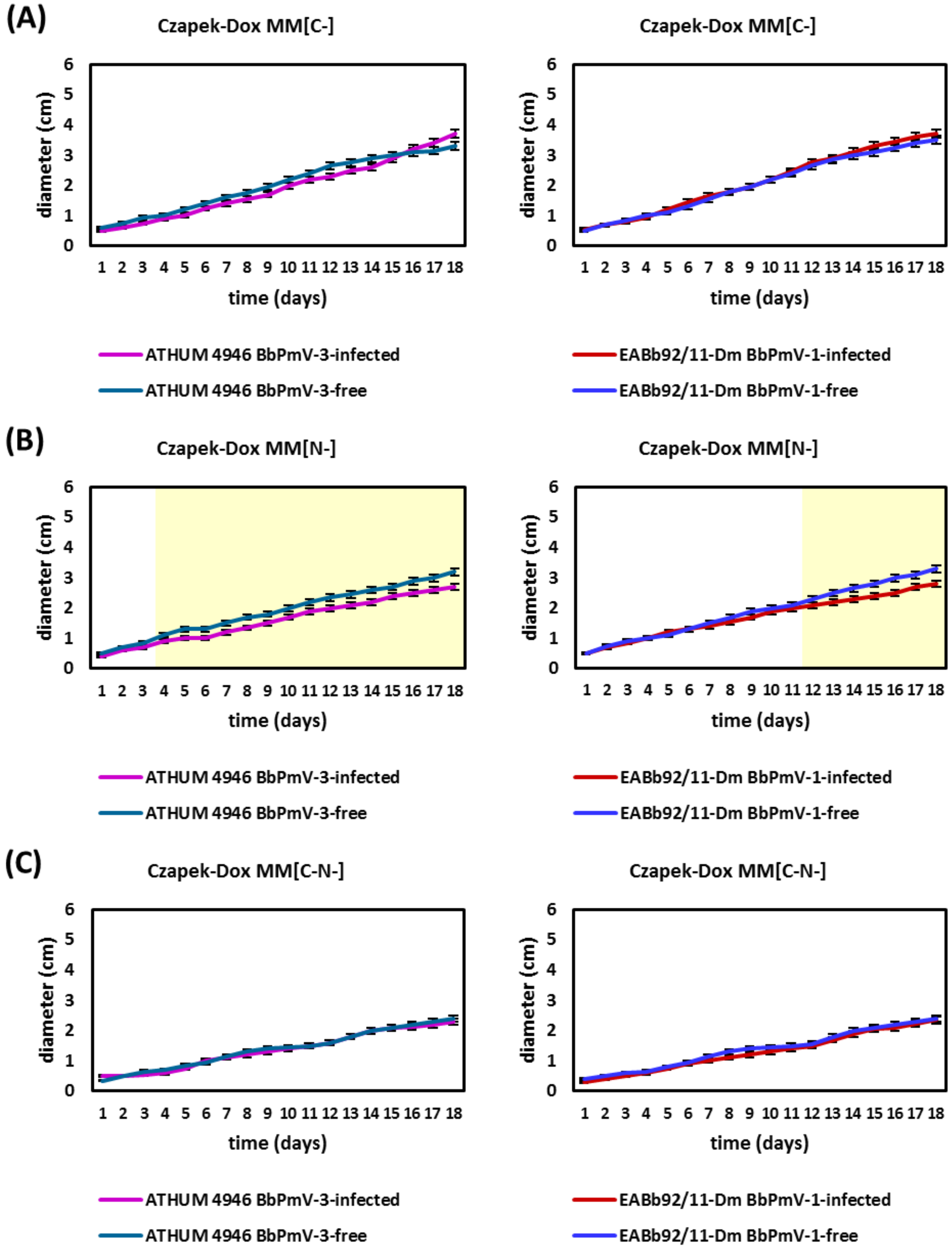

Fig. S9. Growth curves of ATHUM 4946 BbPmV-3–infected and –free (left) and EABb 92/11-Dm BbPmV-1–infected and –free (right) on **(A)** Czapek-Dox MM lacking a carbon source; **(B)** Czapek-Dox MM lacking a nitrogen source; **(C)** Czapek-Dox MM lacking both a carbon and a nitrogen source. Light yellow background indicates a statistically significant difference between the isogenic lines (2-way ANOVA; P-value < 0.05 at least).

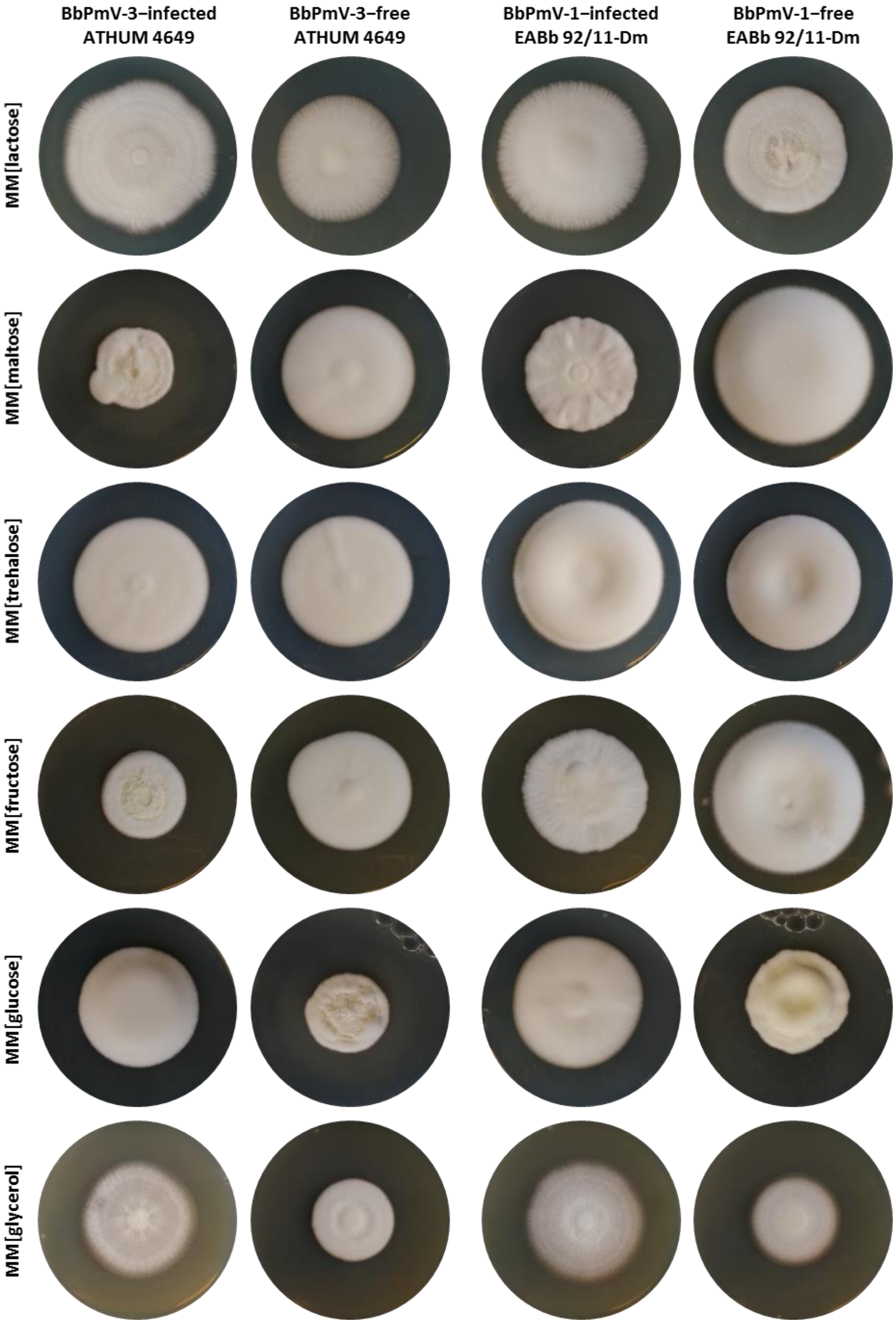

Fig. S10. Cultures of ATHUM 4946 BbPmV-3–infected and –free (left) and EABb 92/11-Dm BbPmV-1–infected and –free (right) on Czapek-Dox MM containing lactose as a carbon source; Czapek-Dox MM containing maltose as a carbon source; Czapek-Dox MM containing trehalose as a carbon source; Czapek-Dox MM containing fructose as a carbon source; Czapek-Dox MM containing glucose as a carbon source; Czapek-Dox MM containing glycerol as a carbon source.

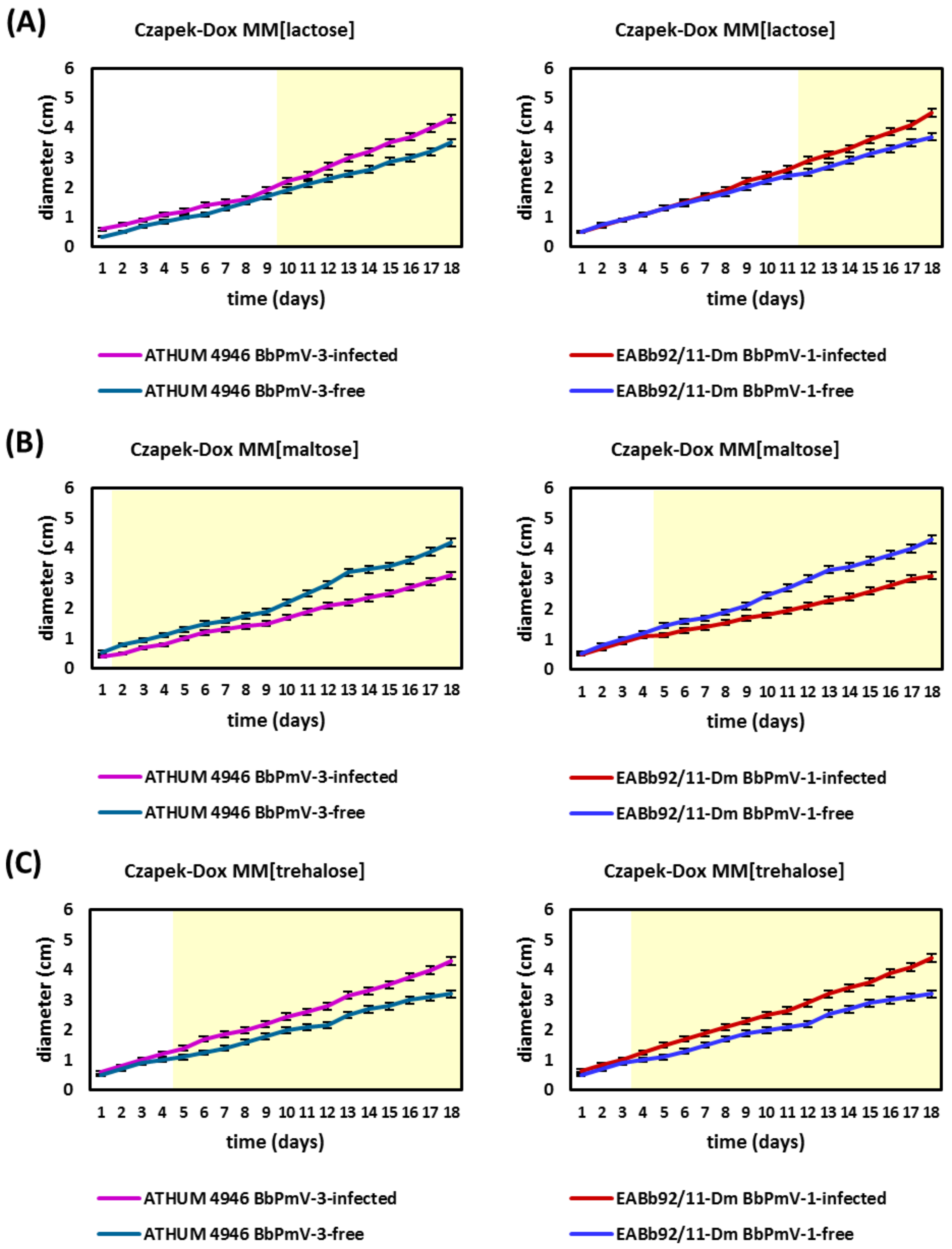

Fig. S11. Growth curves of ATHUM 4946 BbPmV-3–infected and –free (left) and EABb 92/11-Dm BbPmV-1–infected and –free (right) on **(A)** Czapek-Dox MM containing lactose as a carbon source; **(B)** Czapek-Dox MM containing maltose as a carbon source; **(C)** Czapek-Dox MM containing trehalose as a carbon source. Light yellow background indicates a statistically significant difference between the isogenic lines (2-way ANOVA; P-value < 0.05 at least).

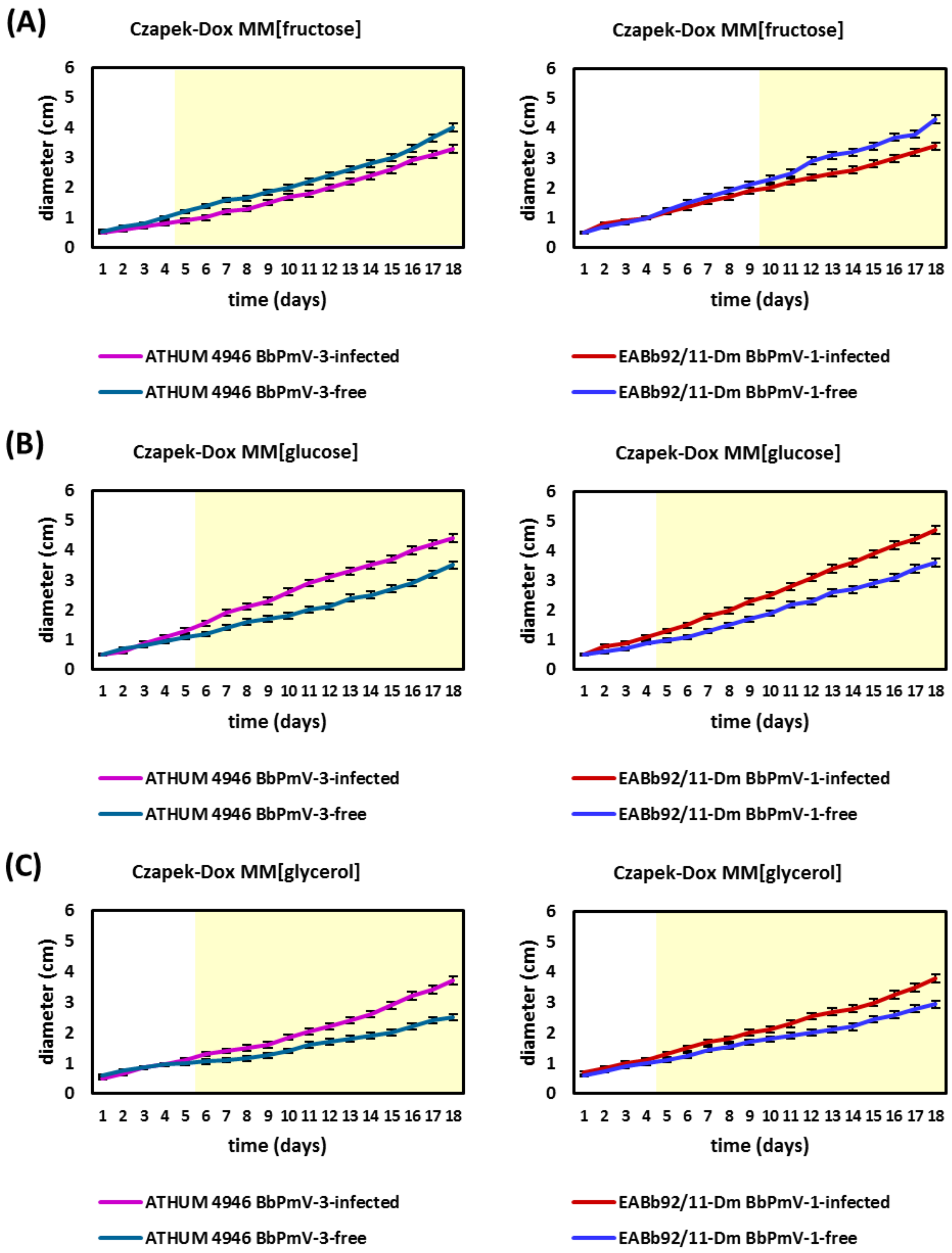

Fig. S12. Growth curves of ATHUM 4946 BbPmV-3–infected and –free (left) and EABb 92/11-Dm BbPmV-1–infected and –free (right) on **(A)** Czapek-Dox MM containing fructose as a carbon source; **(B)** Czapek-Dox MM containing glucose as a carbon source; **(C)** Czapek-Dox MM containing glycerol as a carbon source. Light yellow background indicates a statistically significant difference between the isogenic lines (2-way ANOVA; P-value < 0.05 at least).

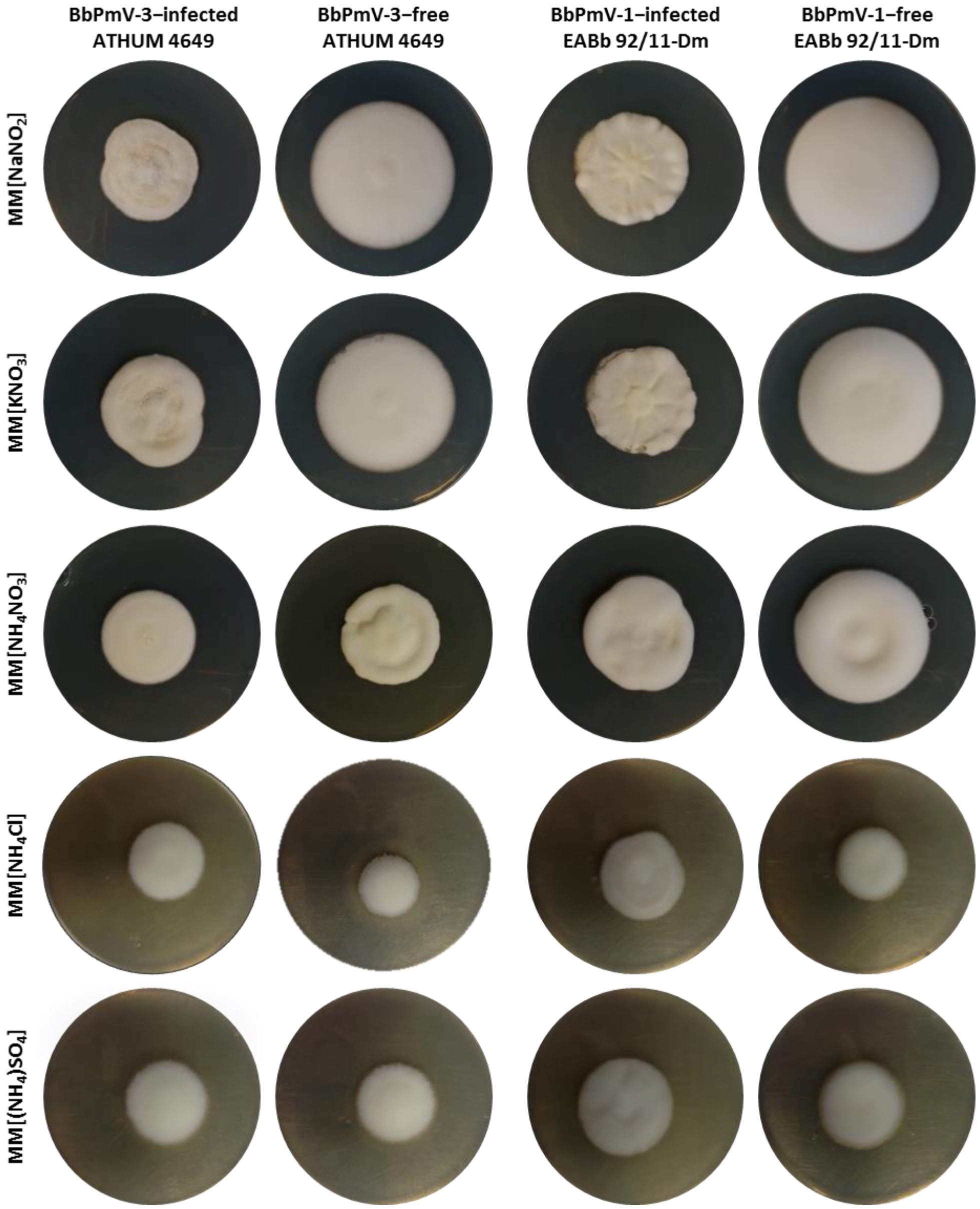

Fig. S13. Cultures of ATHUM 4946 BbPmV-3–infected and –free (left) and EABb 92/11-Dm BbPmV-1–infected and –free (right) on Czapek-Dox MM containing sodium nitrite as a nitrogen source; Czapek-Dox MM containing potassium nitrate as a nitrogen source; Czapek-Dox MM containing ammonium nitrate as a nitrogen source; Czapek-Dox MM containing ammonium chloride as a nitrogen source; Czapek-Dox MM containing ammonium sulfate as a nitrogen source.

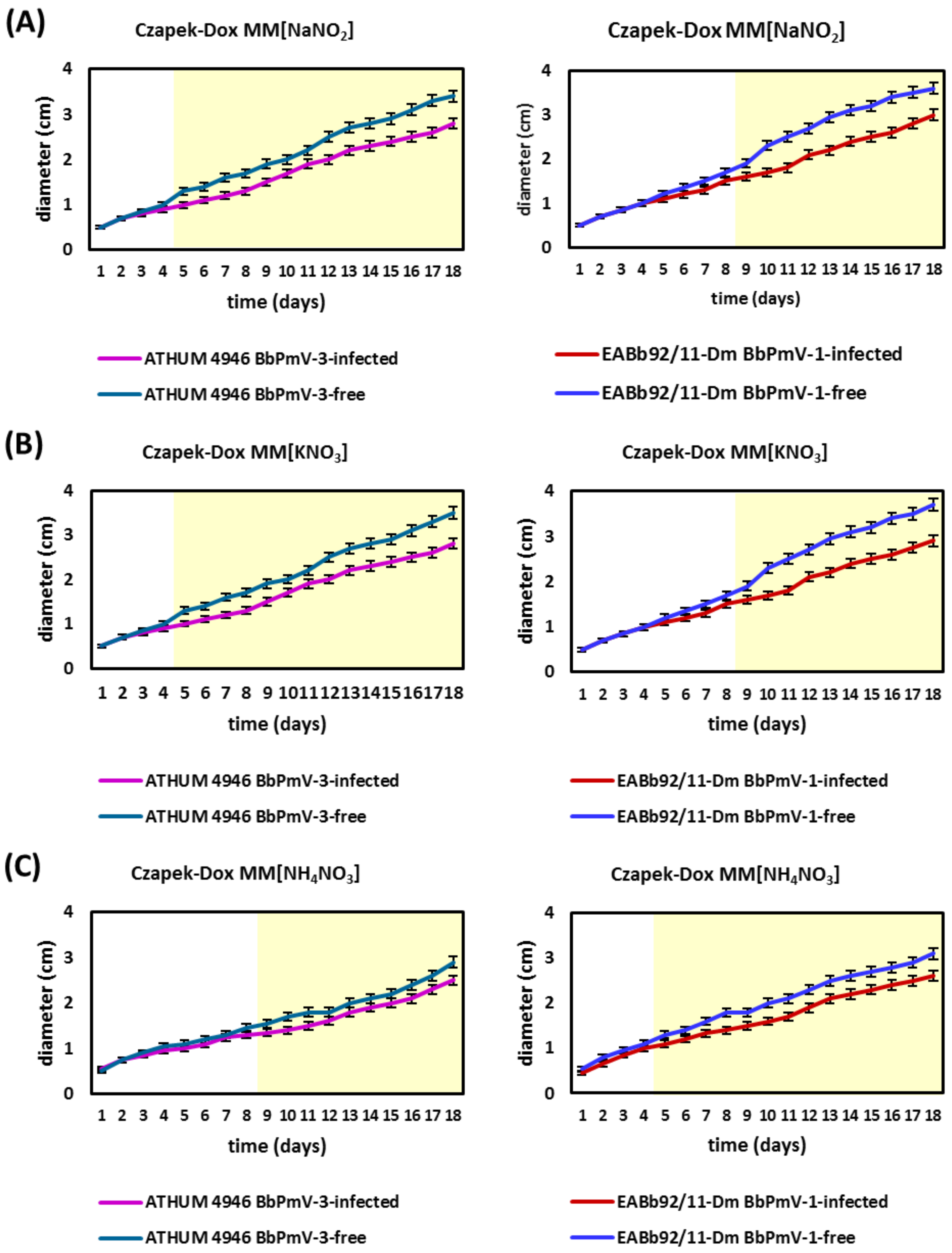

Fig. S14. Growth curves of ATHUM 4946 BbPmV-3–infected and –free (left) and EABb 92/11-Dm BbPmV-1–infected and –free (right) on **(A)** Czapek-Dox MM containing sodium nitrite as a nitrogen source; **(B)** Czapek-Dox MM containing potassium nitrate as a nitrogen source; **(C)** Czapek-Dox MM containing ammonium nitrate as a nitrogen source. Light yellow background indicates a statistically significant difference between the isogenic lines (2-way ANOVA; P-value < 0.05 at least).

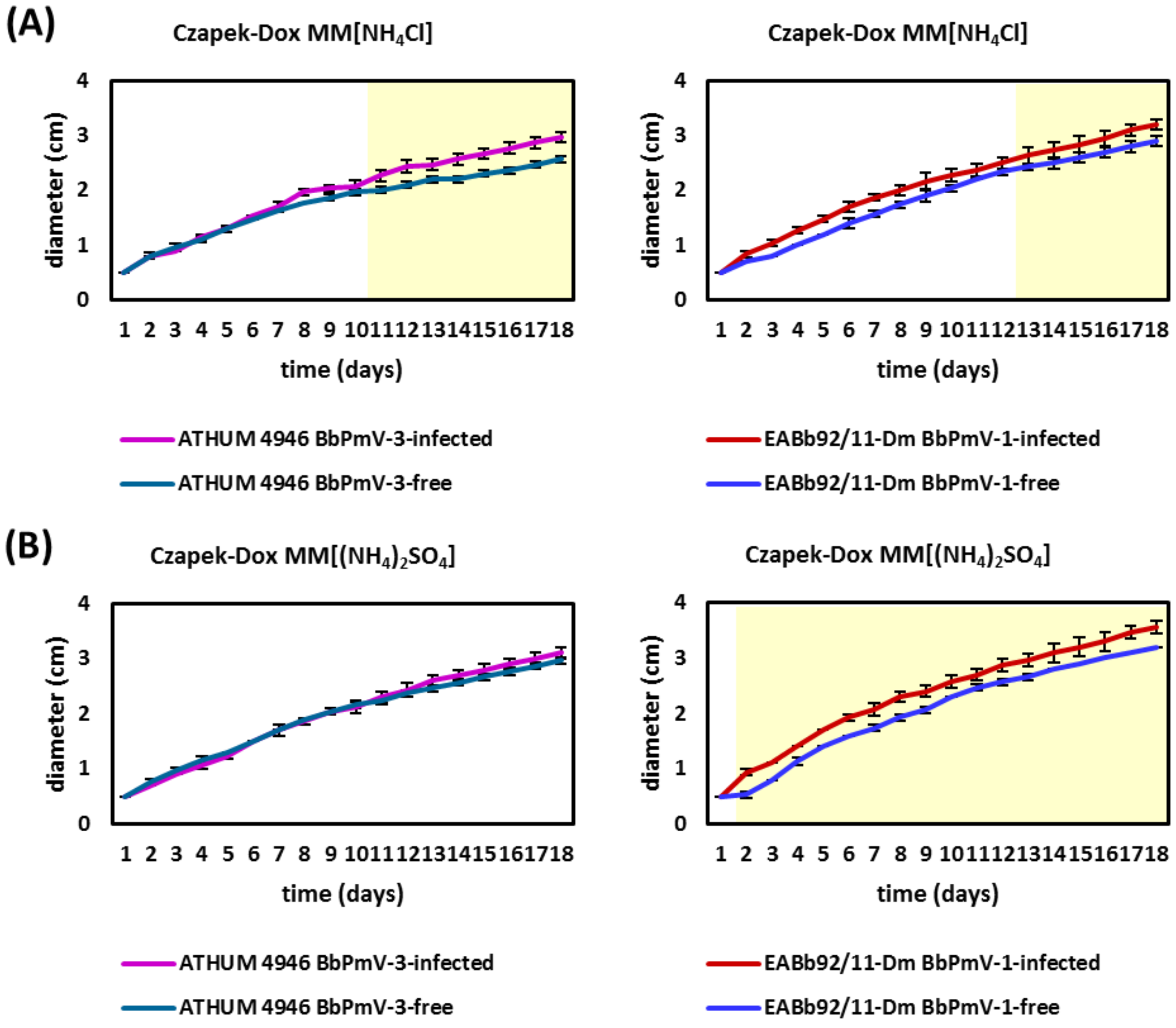

Fig. S15. Growth curves of ATHUM 4946 BbPmV-3–infected and –free (left) and EABb 92/11-Dm BbPmV-1–infected and –free (right) on **(A)** Czapek-Dox MM containing ammonium chloride as a nitrogen source; **(B)** Czapek-Dox MM containing ammonium sulfate as a nitrogen source. Light yellow background indicates a statistically significant difference between the isogenic lines (2-way ANOVA; P-value < 0.05 at least).

Table S1. Polymycoviruses and related viruses.

Virus	Accession number					Isolate	Location (Country)	Ref.
	dsRNA1	dsRNA2	dsRNA3	dsRNA4*	others			
Alternaria tenuissima virus	KP067914	-	-	-	-	Ruzyne312	Czech Republic	unpublished
Alternaria sp. FA0703 sequence	-	FJ595830	-	-	-	FA0703	China	unpublished
Aspergillus fumigatus tetramycovirus 1	HG975302	HG975303	HG975304	HG975305	-			Kanhayuwa *et al*., 2015
Aspergillus fumigatus polymycovirus 1	MH192993	MH192994	MH192995	MH192996	-	V181-30	Netherlands	Zoll *et al*., 2018
Aspergillus spelaeus tetramycovirus 1	MG887754	MG887755	MG887756	MG887757	-	MUT1993	Italy	Nerva *et al*., 2019a
Beauveria bassiana polymycovirus 1	LN896307	LN896308	LN896309	LN896310	-	EABb 92/11-Dm	Spain	Kotta-Loizou & Coutts, 2017
Beauveria bassiana polymycovirus 2	LN896311	-	-	-	5: -<br>6: LN896312<br>7: LN896313	IMI 391043	Syria	Kotta-Loizou & Coutts, 2017
Botryosphaeria dothidea RNA virus 1	KP245734	KP245735	KP245736	KP245737	5: KP245738	YZN115	China	Zhai *et al*., 2016
Cladosporium cladosporioides virus 1	KJ787686	KJ787687	KJ787688	KJ787689	5: KJ787690	DF15	China	unpublished
Colletotrichum camelliae filamentous virus	KX778766	KX778767	KX778768	KX778769	5: KX778770<br>6: KX778771<br>7: KX778772<br>8: KX778773	LT-3-1	China	Jia *et al*., 2017
Fusarium redolens polymycovirus 1	MK609920	MK609921	MK609922	MK609923	5: MK609924<br>6: MK609925<br>7: MK609926<br>8: MK609927	FRPV.A63-1	Belgium	Mahillon *et al*., 2019
Hadaka virus 1	LC519840	LC519841	LC519842	-	4: LC519843*<br>5: LC519844<br>6: LC519845<br>7: LC519846<br>8: LC519847<br>9: LC519848<br>10: LC519849<br>11: LC519850	*Fusarium oxysporum* 7n	Pakistan	Sato *et al*., 2020
Magnaporthe oryzae polymycovirus 1	MH231406	MH231407	MH231408	MH231409	-	TM02	China	unpublished
Melampsora lini sequences	-	unpublished	X64371	-	-	SP6	Australia	Dickinson *et al*. 1993
Mycovirus M7	MN418003	-	-	-	-	-	Colombia	unpublished
Penicillium brevicompactum tetramycovirus 1	MG887750	MG887751	MG887752	MG887753	-	MUT1097	Italy	Nerva *et al*., 2019a
Penicillium digitatum polymycovirus 1	MF317878	MF317879	MF317880	MF317881	-	A	China	Niu *et al*., 2018
Penicillium janthinellum polymycovirus 1	LC571078	LC571079	LC571080	LC571082	4: LC571081*	A58	Pakistan	unpublished
Phaeoacremonium minimum tetramycovirus 1	MK584824	MK584825	MK584826	MK584827	-	CREA-VE-28SY2	Italy	Nerva *et al*., 2019b
Plasmopara viticola lesion associated polymycovirus 1	MN557029	-	MN557030	-	-	DMS6_DN36745	Spain	unpublished
Plasmopara viticola lesion associated polymycovirus 2	MN557032	-	MN557031	MN557033	-	DMS10_DN21785	Spain	unpublished
Plasmopara viticola lesion associated polymycovirus 3	MN557034	-	-	-	-	DMS6_DN31832	Spain	unpublished
Plasmopara viticola lesion associated polymycovirus 4	MN557035	-	-	-	-	DMS5_DN27811	Spain	unpublished
Plasmopara viticola lesion associated polymycovirus 5	MN557036	MN557037	-	MN557038	-	DMG-B_DN52396	Italy	unpublished
Sclerotinia sclerotiorum tetramycovirus 1	MF444217	MF444218	MF444219	-	-	SstRV1S1	Australia	Mu *et al*., 2018

* In all cases, dsRNA1 encodes the RdRP, dsRNA2 the putative scaffold protein and dsRNA3 the methyl-transferase; dsRNA4 encodes the PASrp with the exception of Hadaka virus 1, which does not have a PASrp homologue, and Penicillium janthinellum polymycovirus 1, whose PASrp is encoded by dsRNA5. Additional dsRNAs encode non-homologous proteins of unknown function.

Dickinson, M.J., Zhang, R., Pryor, A. (1993). Nucleotide sequence relationships of double-stranded RNAs in flax rust, *Melampsora lini*. Curr. Genet. 24, 428-432.

Jia, H., Dong, K., Zhou, L., Wang, G., Hong, N., Jiang, D., Xu, W. (2017). A dsRNA virus with filamentous viral particles. Nat. Commun. 8, 168.

Kanhayuwa, L., Kotta-Loizou, I., Özkan, S., Gunning, A.P., Coutts, R.H.A. (2015). A novel mycovirus from *Aspergillus fumigatus* contains four unique dsRNAs as its genome and is infectious as dsRNA. Proc. Natl. Acad. Sci. U. S. A. 112, 9100-9105.

Kotta-Loizou, I., Coutts, R.H.A. (2017). Studies on the virome of the entomopathogenic fungus *Beauveria bassiana* reveal novel dsRNA elements and mild hypervirulence. PLoS Pathog. 13, e1006183.

Mahillon, M., Decroës, A., Liénard, C., Bragard, C., Legrève, A. (2019). Full genome sequence of a new polymycovirus infecting *Fusarium redolens*. Arch. Virol. 164, 2215-2219.

Mu, F., Xie, J., Cheng, S., You, M.P., Barbetti, M.J., Jia, J., Wang, Q., Cheng, J., Fu, Y., Chen, T., Jiang, D. (2018). Virome characterization of a collection of *S. sclerotiorum* from Australia. Front. Microbiol. 8, 2540.

Nerva, L., Forgia, M., Ciuffo, M., Chitarra, W., Chiapello, M., Vallino, M., Varese, G.C., Turina, M. (2019a). The mycovirome of a fungal collection from the sea cucumber *Holothuria polii*. Virus Res. 273, 197737.

Nerva, L., Turina, M., Zanzotto, A., Gardiman, M., Gaiotti, F., Gambino, G., Chitarra, W. (2019b). Isolation, molecular characterization and virome analysis of culturable wood fungal endophytes in esca symptomatic and asymptomatic grapevine plants. Environ. Microbiol. 21, 2886-2904.

Niu, Y., Yuan, Y., Mao, J., Yang, Z., Cao, Q., Zhang, T., Wang, S., Liu, D. (2018). Characterization of two novel mycoviruses from *Penicillium digitatum* and the related fungicide resistance analysis. Sci. Rep. 8, 5513.

Sato, Y., Shamsi, W., Jamal, A., Bhatti, M.F., Kondo, H., Suzuki, N. (2020). Hadaka virus 1: a capsidless eleven-segmented positive-sense single-stranded RNA virus from a phytopathogenic fungus, *Fusarium oxysporum*. mBio 11, e00450-20.

Zhai, L., Xiang, J., Zhang, M., Fu, M., Yang, Z., Hong, N., Wang, G. (2016). Characterization of a novel double-stranded RNA mycovirus conferring hypovirulence from the phytopathogenic fungus *Botryosphaeria dothidea*. Virology 493, 75-85.

Zoll, J., Verweij, P.E., Melchers, W.J.G. (2018). Discovery and characterization of novel *Aspergillus fumigatus* mycoviruses. PLoS One 13, e0200511.

Table S2. Czapek-Dox MM and variations.

Name	Ingredients															
	carbon source (30 g/l)								nitrogen source (3 g/l)							others
	sucrose	fructose	glucose	lactose	maltose	trehalose	glycerol	none	$NaNO_3$	$NaNO_2$	KNO_3	NH_4NO_3	NH_4Cl	$(NH4)_2SO_4$	none	1.0 g/l KH_2PO_4<br>0.5 g/l KCl<br>0.5 g/l $MgCl_2$<br>0.01 g/l $FeSO_4$
MM	+	–	–	–	–	–	–	–	+	–	–	–	–	–	–	+
MM[fructose]	–	+	–	–	–	–	–	–	+	–	–	–	–	–	–	+
MM[glucose]	–	–	+	–	–	–	–	–	+	–	–	–	–	–	–	+
MM[lactose]	–	–	–	+	–	–	–	–	+	–	–	–	–	–	–	+
MM[maltose]	–	–	–	–	+	–	–	–	+	–	–	–	–	–	–	+
MM[trehalose]	–	–	–	–	–	+	–	–	+	–	–	–	–	–	–	+
MM[glycerol]	–	–	–	–	–	–	+	–	+	–	–	–	–	–	–	+
MM[C^-]	–	–	–	–	–	–	–	+	+	–	–	–	–	–	–	+
MM[$NaNO_2$]	+	–	–	–	–	–	–	–	–	+	–	–	–	–	–	+
MM[KNO_3]	+	–	–	–	–	–	–	–	–	–	+	–	–	–	–	+
MM[NH_4NO_3]	+	–	–	–	–	–	–	–	–	–	–	+	–	–	–	+
MM[NH_4Cl]	+	–	–	–	–	–	–	–	–	–	–	–	+	–	–	+
MM[$(NH4)_2SO_4$]	+	–	–	–	–	–	–	–	–	–	–	–	–	+	–	+
MM[N^-]	+	–	–	–	–	–	–	–	–	–	–	–	–	–	+	+
MM[C^-N^-]	–	–	–	–	–	–	–	+	–	–	–	–	–	–	+	+
